# A hepatic amino acid/mTOR/S6K-dependent signalling pathway modulates systemic lipid metabolism via neuronal signals

**DOI:** 10.1038/ncomms8940

**Published:** 2015-08-13

**Authors:** Kenji Uno, Tetsuya Yamada, Yasushi Ishigaki, Junta Imai, Yutaka Hasegawa, Shojiro Sawada, Keizo Kaneko, Hiraku Ono, Tomoichiro Asano, Yoshitomo Oka, Hideki Katagiri

**Affiliations:** 1 Department of Metabolism and Diabetes, Tohoku University Graduate School of Medicine, Sendai 980-8575, Japan; 2 Division of Diabetes and Metabolism, Iwate Medical University, Morioka 020-8505, Japan; 3 The Fourth Department of Internal Medicine, Saitama Medical University, Saitama 350-0495, Japan; 4 Department of Medical Science, Graduate School of Medicine, University of Hiroshima, Hiroshima 734-8553, Japan; 5 Japan Science and Technology Agency, CREST, Sendai 980-8575, Japan

## Abstract

Metabolism is coordinated among tissues and organs via neuronal signals. Levels of circulating amino acids (AAs), which are elevated in obesity, activate the intracellular target of rapamycin complex-1 (mTORC1)/S6kinase (S6K) pathway in the liver. Here we demonstrate that hepatic AA/mTORC1/S6K signalling modulates systemic lipid metabolism via a mechanism involving neuronal inter-tissue communication. Hepatic expression of an AA transporter, SNAT2, activates the mTORC1/S6K pathway, and markedly elevates serum triglycerides (TGs), while downregulating adipose lipoprotein lipase (LPL). Hepatic Rheb or active-S6K expression have similar metabolic effects, whereas hepatic expression of dominant-negative-S6K inhibits TG elevation in SNAT2 mice. Denervation, pharmacological deafferentation and β-blocker administration suppress obesity-related hypertriglyceridemia with adipose LPL upregulation, suggesting that signals are transduced between liver and adipose tissue via a neuronal pathway consisting of afferent vagal and efferent sympathetic nerves. Thus, the neuronal mechanism uncovered here serves to coordinate amino acid and lipid levels and contributes to the development of obesity-related hypertriglyceridemia.

## Abstract (web summary)

Neuronal signals can coordinate metabolic processes across tissues. Here, the authors show that plasma amino acid and triglyceride levels are linked by a neuronal mechanism that couples amino acid sensing in the liver with the expression of lipoprotein lipase in adipose tissue.

## Graphical abstract

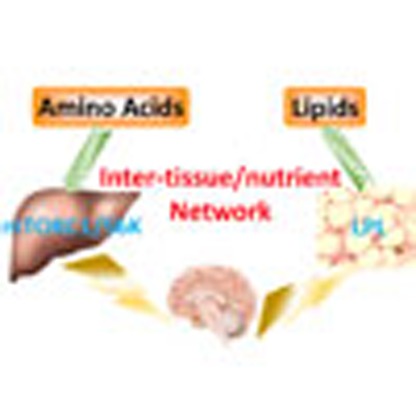

Metabolism in different tissues/organs is considered to be regulated in a coordinated manner systemically, thereby allowing adaptation to a variety of environmental conditions^1^,^2^. In addition to humoral factors, such as insulin and adipokines, neuronal signals have recently attracted increasing attention for their roles in this inter-tissue metabolic harmony^1^. In particular, the neuronal relay systems originating in the liver have been identified as playing important roles in modulating energy^3^ and glucose^4^ metabolism by enhancing energy expenditure and increasing pancreatic β-cell mass, respectively. Furthermore, another neuronal relay system, originating in alterations in hepatic glucose metabolism, was recently reported to function as an energy-saving mechanism at the whole-body level^5^.

Herein, we focused on the effects of hepatic amino acid (AA) signalling on systemic regulation of metabolism. Although intake of surplus energy, such as increased fat, is widely considered to be a major cause of metabolic derangements, protein overload also reportedly plays a role in obesity-related metabolic disorders^6^,^7^. Circulating AAs, mainly from dietary protein, enter the cytoplasm via AA transporters (AATs), which are responsible for the first step in AA signalling^8^. Intracellular AAs activate the mammalian target of rapamycin (mTOR) complex-1 (mTORC1)/S6kinase (S6K) pathway via several signalling cascades^7^,^9^,^10^. In addition to this signalling cascade from AAs, other intracellular or extracellular cues, such as growth factors, oxygen, stress and energy levels, also function to regulate this mTORC1 pathway, leading to the activation of several downstream targets, including S6K^11^,^12^. The mTORC1 signalling pathway has been recognized as the major controller of many important cellular processes, for example, protein translation, lipid synthesis, stress response and autophagy, the deregulation of which occurs in human diseases including cancer, obesity and type 2 diabetes^11^,^12^. In obese subjects, serum AAs are reportedly elevated^6^,^13^,^14^ and the mTORC1/S6K pathway is activated in organs including the liver^15^,^16^. The hepatic mTORC1 pathway is reportedly activated in obese or aged states resulting in the development of hepatic steatosis^17^,^18^,^19^ and suppression of fasting-induced ketogenesis^20^, indicating important roles in the systemic metabolism disturbances that are associated with obesity development. However, little is known about the roles of hepatic mTORC1/S6K activation in obese states and the metabolic relationships between the liver and other organs/tissues during obesity development.

In the present study, to increase AA entry into hepatocytes, we expressed an AAT, SNAT2, in the murine liver using the adenoviral gene delivery system. Hepatic SNAT2 expression activated the mTORC1/S6K pathway, leading to elevation of serum triglyceride (TG) levels, especially in postprandial states. Decreased plasma activity of TG hydrolysis associated with adipose lipoprotein lipase (LPL) downregulation is responsible for the observed hypertriglyceridemia. This inter-tissue mechanism is mediated by a neuronal pathway from the liver. Therefore, this novel inter-nutrient and inter-tissue communication mechanism originating in the hepatic AA/mTORC1/S6K pathway modulates systemic lipid metabolism and makes an important contribution to the development of obesity-related hypertriglyceridemia.

## Results

### Hepatic SNAT2 expression activates mTORC1/S6K pathway

**Figure 1:**
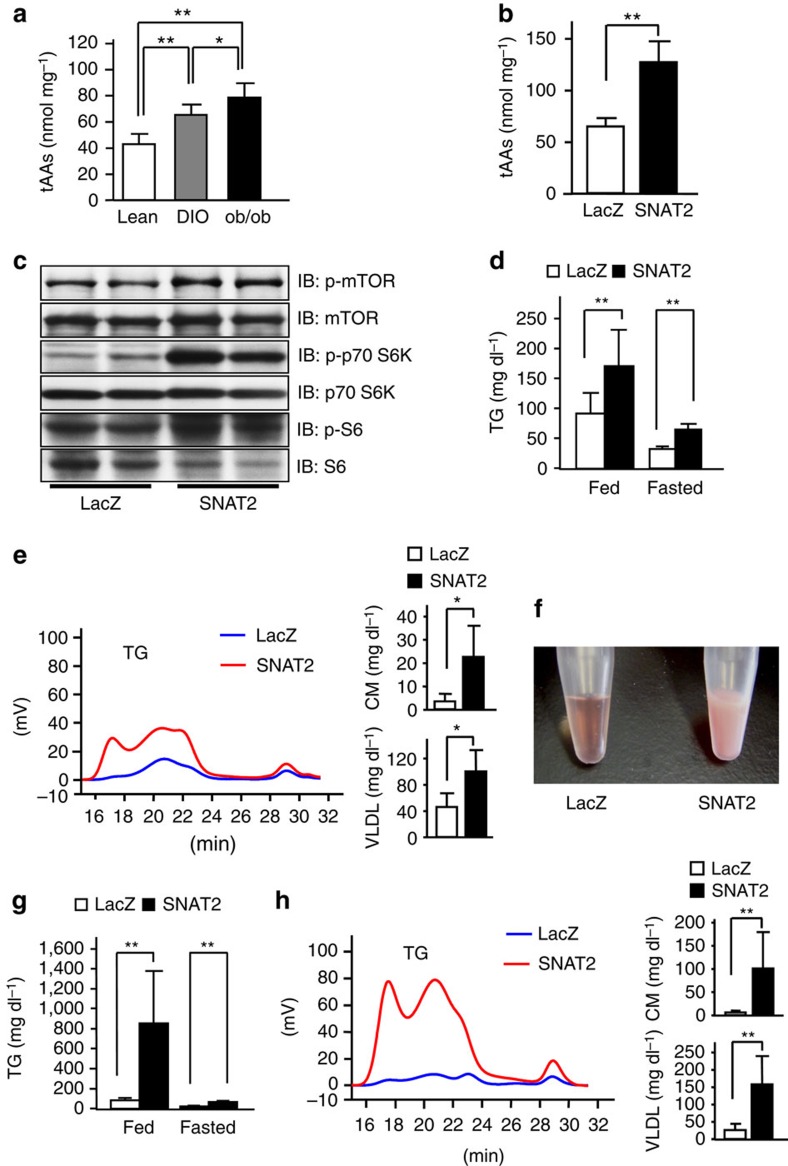
Hepatic SNAT2 expression activates mTORC1/S6K pathway and induces hypertriglyceridemia. (**a**) Hepatic total AA concentrations were measured in 8-week-old lean (white bar) or DIO (grey bar) and ob/ob (black bar) mice (*n*=5–7). (**b**–**h**) SNAT2 (black bars) or control LacZ (white bars) adenovirus was administered to high-fat diet-fed C57BL/6 mice (**b**, **c** and **f**–**h**), and standard chow-fed C57BL/6 mice (**d** and **e**). (**b**) Total AA concentrations in the liver were measured (*n*=5). (**c**) Immunoblotting of liver extracts with anti-phospho-mTOR, mTOR, phospho-p70-S6K, p70-S6K, phospho-S6 and S6 antibodies. The representative images derived from at least two experiments were displayed. (**d** and **g**) Serum TG levels under 10 h-fasted or -fed conditions were measured (**d**; *n*=5–7, **g**; *n*=5–7) and (**e** and **h**) HPLC analyses of sera in fed states were performed on day 5 after adenovirus administration (**e**; *n*=6–7, **h**; *n*=5–6). (**f**) Photograph of sera obtained from controls (left) and SNAT2 mice (right) fed a high-fat diet. Data are presented as means±s.d. *P<0.05, **P<0.01 by the unpaired *t*-test.

AA entry into cells is well known to activate the mTORC1/S6K pathway^7^,^9^,^10^,^12^ and the mTOR/S6K pathway in the liver is reportedly activated in obese subjects^15^,^16^. Therefore, in this study, we focused on the role of the hepatic AA/mTORC1/S6K pathway in metabolic derangements associated with obesity. First, we found elevation of hepatic total AA concentrations in obesity models, that is, mice with high-fat diet-induced obesity (DIO) and genetically obese ob/ob mice (Fig. 1a), suggesting increased AA entry into the liver in the obese state.

Therefore, we aimed to elucidate the roles of increased AA entry into the liver in systemic metabolism. To increase AA entry into the liver, we expressed an AAT, SNAT2 (slc38a2)^8^, which transports neutral AAs, in the liver (SNAT2 mice) using adenoviral gene transduction. Systemic infusion of SNAT2 recombinant adenovirus resulted in selective transgene expression in the liver (Supplementary Fig. 1a,b) without increasing expressions in other tissues, as reported previously^3^,^4^. Mice given the lacZ adenovirus were used as controls^21^. Hepatic SNAT2 expression significantly increased hepatic total AA concentrations (Fig. 1b), which were considered to be within the physiological range, in comparison with reported AA levels after high-fat loading for 16 weeks^22^. Hepatic concentrations of a number of AAs, especially neutral AAs, were increased in SNAT2 mice (Supplementary Table 1). Furthermore, the mTORC1/S6K pathway was actually activated in the livers of SNAT2 mice (Fig. 1c). Activation of the mTORC1/S6K pathway reportedly increases insulin-stimulated serine phosphorylation of insulin receptor substrates, leading to the blockade of insulin signalling. In fact, insulin-stimulated serine473 phosphorylation of Akt was decreased in the livers of SNAT2 mice (Supplementary Fig. 2). Thus, this transporter was functionally expressed and enhanced mTORC1/S6K signalling in the liver.

### Hepatic SNAT2 expression induces hypertriglyceridemia

In wild-type mice fed standard chow, hepatic SNAT2 expression significantly raised serum TGs, especially in the chylomicron and very low density lipoprotein (VLDL) fractions (Fig. 1d,e) in fed states. Furthermore, when we expressed SNAT2 in the livers of DIO mice, to our surprise, sera from DIO-SNAT2 mice had a remarkable milky-white appearance (Fig. 1f). Serum TGs were markedly elevated, especially in fed states (Fig. 1g). TGs in the chylomicron and VLDL fractions were increased in SNAT2 mice (Fig. 1h). Thus, hepatic AA signalling affects systemic lipid metabolism, particularly in postprandial states. High-fat feeding enhanced the TG-elevating effects in SNAT2 mice.

### Hepatic SNAT2 expression suppresses TG breakdown

**Figure 2:**
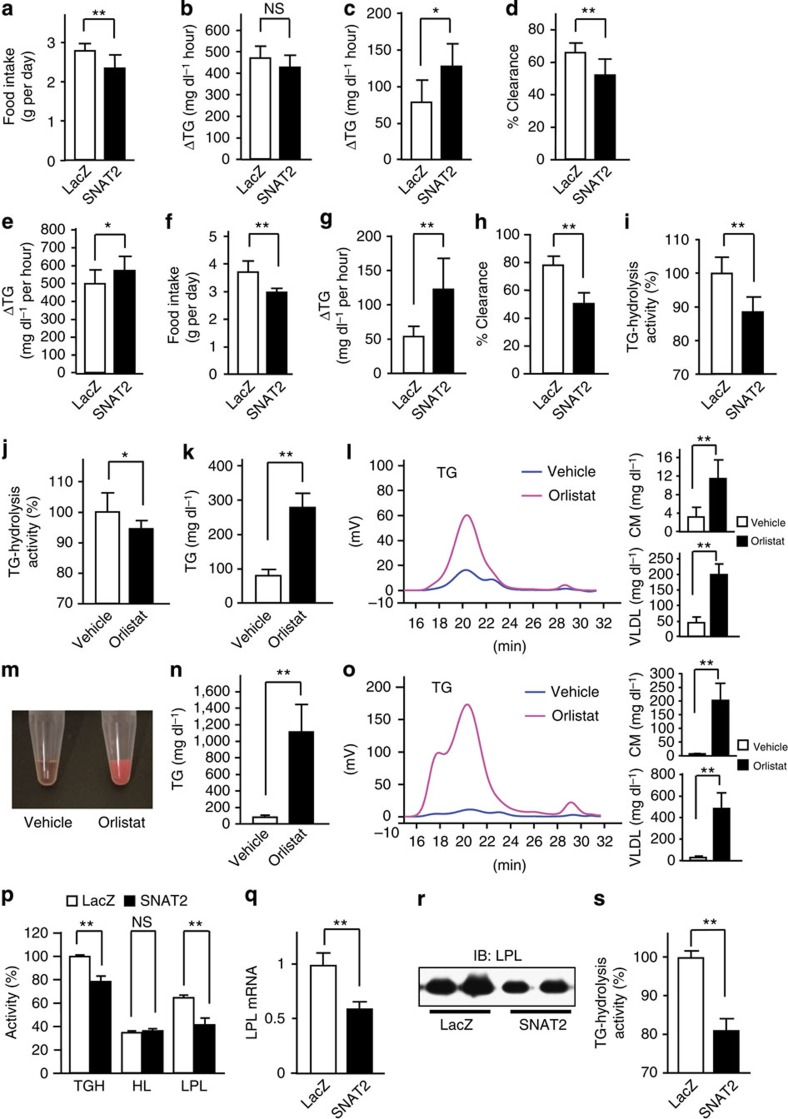
Suppression of plasma TG-hydrolysis activity induces hypertriglyceridemia. SNAT2 (black bars) or control LacZ (white bars) adenovirus was administered to standard chow-fed C57BL/6 mice (**a**–**d**), or high-fat diet-fed C57BL/6 mice (**e**–**i**). (**a** and **f**) Food intakes were measured for 5 days after adenovirus administration (**a**; *n*=11, **f**; *n*=5–6). (**b** and **e**) The rates of hepatic TG secretion were determined (**b**; *n*=5–6, **e**; *n*=9–11). (**c** and **g**) Fat-tolerance tests were performed (**c**; *n*=4–8, **g**; *n*=3–7). (**d** and **h**) TG-clearance studies were performed (**d**; *n*=5–11, **h**; *n*=5–6). (**i**) Plasma TG-hydrolysis activities were measured after the injection of heparin into the tail veins of 4 h-fasted mice (*n*=4–5). (**j**–**l**) Vehicle (white bars) or orlistat (black bars) was administered into the intraperitoneal space of C57BL/6 mice fed standard chow. Plasma TG-hydrolysis activities (**j**) were measured 90 min after the injection of these agents (*n*=7–8). Next, serum TG levels (**k**) were measured, and HPLC analyses of sera from these mice (**l**) were performed (**k**; *n*=5, **l**; *n*=5). (**m**–**o**) Vehicle (white bars) or orlistat (black bars) was administered into the intraperitoneal space of C57BL/6 mice fed a high-fat diet. (**m**) Photograph of sera obtained from vehicle- (left) and orlistat-administered mice (right). Serum TG levels (**n**) were measured, and HPLC analyses of sera from these mice (**o**) were performed (**n**; *n*=4–5, **o**; *n*=4). (**p**–**s**) SNAT2 (black bars) or control LacZ (white bars) adenovirus was administered to high-fat diet-fed C57BL/6 mice. (**p**) TG-hydrolysis activities were measured at either a normal or a high NaCl concentration after the injection of heparin into the tail veins of 4 h-fasted mice (*n*=6). (**q**) LPL mRNA expressions in WAT were analysed by RT–PCR (*n*=5–7). (**r**) Immunoblotting of WAT extracts with anti-LPL antibody. The representative images derived from at least two experiments were displayed. (**s**) TG-hydrolysis activity in WAT was examined on day 5 after adenovirus administration (*n*=5). Data are presented as means±s.d. *P<0.05, **P<0.01 by the unpaired *t*-test. NS, not significant.

We sought to determine whether the mechanism raising serum TGs involves increased TG production or decreased TG breakdown. Dietary fats are hydrolysed to monoacylglycerol and fatty acids in the intestinal lumen, and then enter the circulation as re-synthesized TGs^23^. The liver also secretes TGs. These TGs are, in turn, hydrolysed into fatty acids by TG lipases, such as LPL and hepatic lipase (HL). In SNAT2 mice fed standard chow, food intakes were not increased, instead was somewhat decreased (Fig. 2a), and hepatic TG secretion rates were not increased (Fig. 2b). Hepatic expression of molecules related to hepatic lipid metabolism, such as sterol regulatory element-binding protein 1c (SREBP1c), microsomal triacylglycerol transfer protein (MTP), LDL receptor (LDLR) and peroxisome proliferator-activated receptor α (PPARα), were unaltered in SNAT2 mice (Supplementary Fig. 3a). Again, hepatic expressions of secreted proteins, which function as regulators of plasma TG hydrolysis, such as apoC-2 and 3, and angiopoietin-like protein (angptl)-3, 4 and 8, and glycosylphosphatidylinositol-anchored high-density lipoprotein-binding protein 1 (GPIHBP1), were also unaltered in lean SNAT2 mice (Supplementary Fig. 3b). In contrast, elevation of serum TGs after olive oil (unsaturated fatty acids) loading, which reportedly reflects decreases in TG breakdown^24^, was markedly enhanced in SNAT2 mice (Fig. 2c). In addition, the clearance of serum TG after injection with soybean oil emulsion, Intralipid, through the tail vein was significantly reduced in SNAT2 mice (Fig. 2d). These findings, taken together, indicate that decreased TG breakdown is responsible for hypertriglyceridemia in SNAT2 mice.

On the other hand, under conditions of obesity, such as in DIO mice, hepatic TG secretion rates of SNAT2 mice were slightly enhanced (Fig. 2e), whereas food intakes were not increased (Fig. 2f). Hepatic expressions of MTP and LDLR were upregulated in DIO-SNAT2 mice (Supplementary Fig. 3c) and hepatic TG contents were elevated in these mice (Supplementary Fig. 3d). Therefore, in the settings of obesity, increased hepatic TG secretion is likely to make a slight contribution to the marked TG elevation in SNAT2 mice. However, the serum TG elevation after olive oil loading was more markedly enhanced by hepatic SNAT2 expression in DIO mice than in normal chow-fed mice (Fig. 2g). Serum TG clearance after Intralipid injection was remarkably reduced in SNAT2-obese mice (Fig. 2h). In fact, hepatic SNAT2 expression significantly decreased plasma TG-hydrolysis activity, by 11.4%, in DIO mice (Fig. 2i). LacZ adenovirus administration alone did not change plasma TG-hydrolysis activity (Supplementary Fig. 4a). These findings suggest that decreased TG breakdown plays an important role in the hypertriglyceridemia in DIO mice as well. This notion is consistent with the finding that high-fat feeding further elevated serum TG levels in postprandial states.

### Suppression of TG-hydrolysis causes hypertriglyceridemia

The next question is whether the slight decrease in TG-hydrolysis activity observed in SNAT2 mice actually induces the hypertriglyceridemia? To examine this possibility, we intraperitoneally administered a lipase inhibitor, orlistat^25^,^26^, to wild-type mice fed standard chow. In fact, 250 mg kg^−1^ body weight orlistat significantly decreased TG-hydrolysis activity (Fig. 2j) in a manner similar to that observed in SNAT2 mice (Fig. 2i). Serum TGs, especially in the chylomicron and VLDL fractions, were actually increased by intraperitoneal orlistat administration (Fig. 2k,l). When orlistat was administered at the same dosage to wild-type mice fed a high-fat diet, sera from these mice exhibited a remarkable milky-white appearance (Fig. 2m), resulting from the marked increase in serum TGs, especially in the chylomicron and VLDL fractions (Fig. 2n,o). In addition, adenoviral expression of angptl-4, known to inhibit plasma LPL activity, in the liver significantly decreased TG-hydrolysis activity (Supplementary Fig. 5a,b) in a manner similar to that observed in SNAT2 mice (Fig. 2i), leading to serum TG elevation, especially in the chylomicron and VLDL fractions, even in lean mice (Supplementary Fig. 5c,d). Thus, the slight decrease in TG-hydrolysis activity alone can explain the induction of hypertriglyceridemia observed in SNAT2 mice under both normal chow- and high-fat-fed conditions. Taken together, these observations indicate that suppression of TG-hydrolysis activity plays a major role in the serum TG elevation in SNAT2 mice.

### Hepatic SNAT2 expression decreases adipose LPL expression

Plasma TG-hydrolysis activity is considered to involve several lipases, including LPL, synthesized by white adipose tissue (WAT), skeletal muscle and cardiac muscle^27^,^28^, and HL from hepatocytes^29^. First, we measured heparinized plasma TG-hydrolysis activity under normal and high-salt conditions to distinguish between LPL and HL activities^30^. This procedure revealed that salt-insensitive TG-hydrolysis activities (by HL) were similar in SNAT2 and control mice, while salt-inhibited TG-hydrolysis activities (by LPL) were significantly decreased (Fig. 2p) in SNAT2 mice, by approximately 30%, the difference being larger than total TG-hydrolysis activity. Thus, it was mainly the decrease in plasma LPL activity that contributed to the reduction in plasma TG-hydrolysis activity observed in SNAT2 mice.

Interestingly, LPL mRNA expression was significantly decreased in WAT of SNAT2 mice (Fig. 2q), while LacZ adenovirus administration alone did not change LPL expression in WAT (Supplementary Fig. 4b). On the contrary, LPL mRNA expressions were not decreased in brown adipose tissue, cardiac muscle and skeletal muscle (Supplementary Fig. 6a). In addition, LPL protein expression was also decreased in WAT of SNAT2 mice (Fig. 2r). Furthermore, TG-hydrolysis activity in WAT, that is, LPL activity in WAT, was significantly decreased (Fig. 2s), while those obtained from both cardiac and skeletal muscles were unaltered (Supplementary Fig. 6b). In contrast, HL expression and TG-hydrolysis activity in the liver, that is, HL activity, were not decreased, instead being somewhat increased, in SNAT2 mice (Supplementary Fig. 6c). Thus, LPL expression, particularly in WAT, is likely to make an important contribution to decreased plasma TG-hydrolysis activity and the resultant hypertriglyceridemia in SNAT2 mice. These findings suggest that alterations in hepatic AA metabolism affect adipose LPL expression, thereby regulating systemic lipid metabolism.

### Hepatic mTORC1/S6K pathway induces hypertriglyceridemia

**Figure 3:**
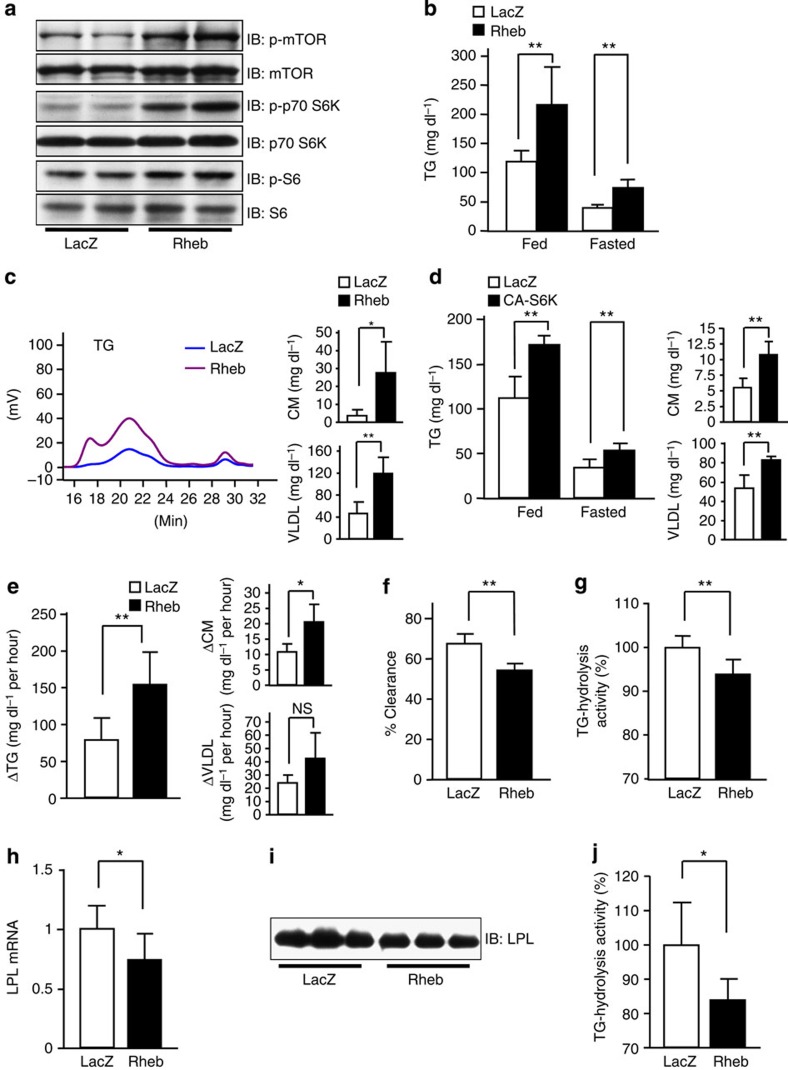
Activation of hepatic mTORC1/S6K pathway leads to hypertriglyceridemia. (**a**–**c**) Rheb (black bars) or LacZ (white bars) adenovirus was administered to C57BL/6 mice. (**a**) Immunoblotting of liver extracts with anti-phospho-mTOR, mTOR, phospho-p70-S6K, p70-S6K, phospho-S6 and S6 antibodies. Serum TG levels under 10 h-fasted or -fed conditions were measured (**b**), HPLC results of sera in fed states were determined (**c**) on day 5 after adenovirus administration (**b**; *n*=5–7, **c**; *n*=4). (**d**) CA-S6K (black bars) or LacZ (white bars) adenovirus was administered to C57BL/6 mice. Serum TG levels under 10 h-fasted or -fed conditions were measured, and HPLC results of sera in fed states were determined on day 5 after adenovirus administration (*n*=4–6). (**e**–**j**) Rheb (black bars) or LacZ (white bars) adenovirus was administered to C57BL/6 mice. (**e**) Fat-tolerance tests were performed, followed by HPLC analysis (*n*=4–8). (**f**) TG-clearance studies were performed (*n*=5–6). (**g**) Plasma TG-hydrolysis activity was measured after the injection of heparin into the tail veins of 4 h-fasted mice (*n*=6–8). (**h**) LPL mRNA expression in WAT was analysed by RT–PCR (*n*=6–7). (**i**) Immunoblotting of WAT extracts with anti-LPL antibody. (**j**) TG-hydrolysis activity in WAT was examined on day 5 after adenovirus administration (*n*=6–7). The representative images derived from at least two experiments were displayed (**a** and **i**). Data are presented as means±s.d. *P<0.05, **P<0.01 by the unpaired *t*-test. NS, not significant.

We next focused on the mechanism whereby hepatic SNAT2 expression suppresses adipose LPL expression. As shown in Fig. 1c, hepatic SNAT2 expression activated mTORC1/S6K signalling in the liver. Therefore, to examine whether hepatic mTOR/S6K activation leads to hypertriglyceridemia, we expressed Rheb, which reportedly activates mTORC1 (refs 9, 10, 31). Adenoviral overexpression of Rheb in the livers of wild-type mice fed standard chow (Rheb mice) (Supplementary Fig. 7a) enhanced phosphorylations of hepatic mTOR, p70-S6K and S6 ribosomal protein (Fig. 3a), while hepatic SNAT2 expression was not increased, instead being somewhat decreased in Rheb mice (Supplementary Fig. 7b). Under these conditions, serum TGs were significantly elevated (Fig. 3b) and TG contents in the chylomicron and VLDL fractions were also increased (Fig. 3c), findings similar to those in SNAT2 mice. As in SNAT2 mice fed standard chow, food intakes and hepatic TG secretion rates were not increased in Rheb mice (Supplementary Fig. 7c,d). In addition, adenoviral overexpression of the constitutively active form of p70-S6kinase (CA-S6K) in the livers of wild-type mice fed standard chow also raised serum TG levels and the TG contents of the chylomicron and VLDL fractions (Fig. 3d). In addition, serum TGs and TG contents of the chylomicron fractions after olive oil loading were both elevated in Rheb mice (Fig. 3e). Serum TG clearance after Intralipid injection was significantly reduced in Rheb mice (Fig. 3f). Indeed, plasma TG-hydrolysis activity as well as LPL expression and TG-hydrolysis activity in WAT were significantly decreased by hepatic Rheb expression (Fig. 3g–j). On the other hand, lipid metabolism-related molecules, such as MTP, LDLR, SREBP1c and PPARα, in the liver were not upregulated in lean Rheb mice (Supplementary Fig. 7e) and hepatic TG contents were not significantly altered (Supplementary Fig. 7f). Hepatic expressions of secreted proteins such as apoC-2 and -3, angptl-3, -4 and -8, and GPIHBP1 were unaltered in lean Rheb mice (Supplementary Fig. 7g). Thus, hepatic mTORC1/S6K activation induces hypertriglyceridemia involving decreased LPL expression in WAT.

### Restoration of TG-hydrolysis reverses hypertriglyceridemia

**Figure 4:**
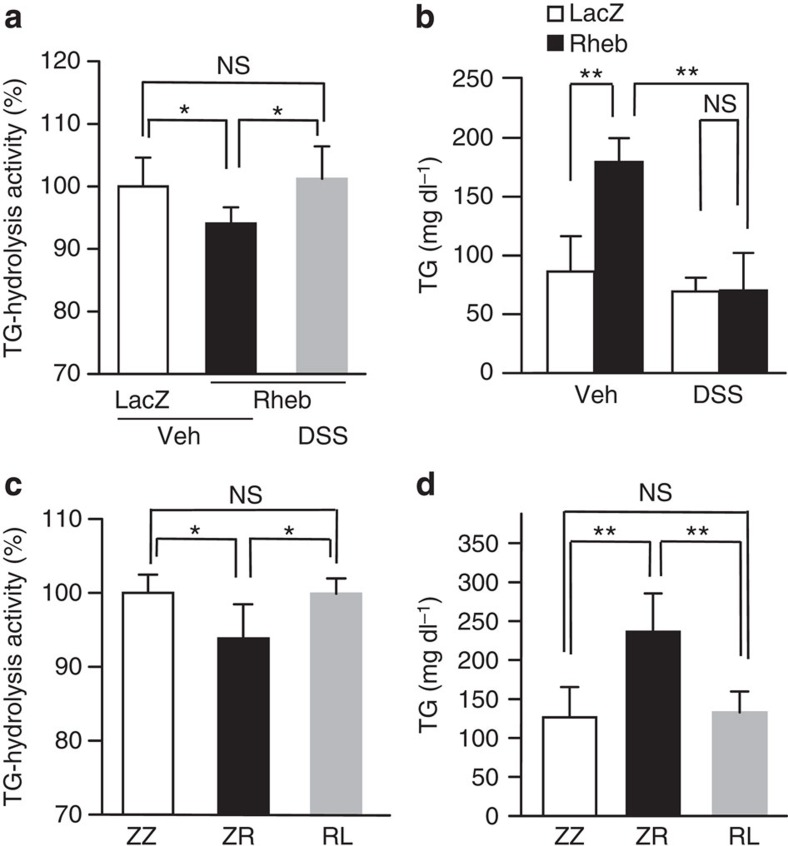
Restoration of plasma TG-hydrolysis activities improves Rheb-induced hypertriglyceridemia. (**a**,**b**) Rheb (black bars) or LacZ (white bars) adenovirus was administered to C57BL/6 mice. Vehicle (Veh) or dextran sodium sulfate (DSS) was intravenously administered to standard chow-fed C57BL/6 mice 5 days after adenoviral administration. Plasma TG-hydrolysis activities (**a**) were measured and serum TG levels (**b**) were examined 30 min after injection of these agents (**a**; *n*=6–7, **b**; *n*=3–4). (**c**,**d**) Mixed adenoviruses, that is, double-dose LacZ-adenovirus (ZZ), LacZ plus Rheb (ZR) and Rheb plus LPL (RL) adenoviruses, were administered to C57BL/6 mice. Plasma TG-hydrolysis activities (**c**) were measured, and serum TG levels (**d**) were examined 5 days after adenoviral administration (**c**; *n*=5–6, **d**; *n*=5–6). Data are presented as means±s.d. *P<0.05, **P<0.01 by the unpaired *t*-test. NS, not significant.

To determine the involvement and significance of LPL in the present phenotype, we examined whether restoration of plasma TG-hydrolysis activity actually does blunt serum TG elevation in Rheb mice. First, we administered dextran sodium sulfate (DSS), an LPL activator, intravenously to lean Rheb mice. DSS administration in Rheb mice restored plasma TG-hydrolysis activities to a degree similar to those observed in LacZ mice (Fig. 4a). Under this condition, serum TG elevation induced by hepatic Rheb expression was almost completely inhibited (Fig. 4b). Next, to compensate for the LPL decrease in Rheb mice, LPL protein was re-expressed using the adenoviral gene expression procedure. Mixed recombinant adenoviruses, Rheb plus LPL (RL mice), LacZ plus Rheb (ZR mice) and double-dose LacZ (ZZ mice), were administered to C57BL/6 mice fed standard chow. We searched for an appropriate adenovirus titre at which plasma TG-hydrolysis activity was increased in Rheb mice in a manner similar to that in LacZ mice (Fig. 4c). Under this condition, Rheb-induced hypertriglyceridemia was markedly improved by LPL expression to an extent similar to that observed in ZZ mice (Fig. 4d). These findings, taken together, further confirmed that decreased plasma TG-hydrolysis activity is responsible for the hypertriglyceridemia induced by mTORC1/S6K activation in the liver.

### SNAT2 induces hypertriglyceridemia via mTORC1/S6K activation

**Figure 5:**
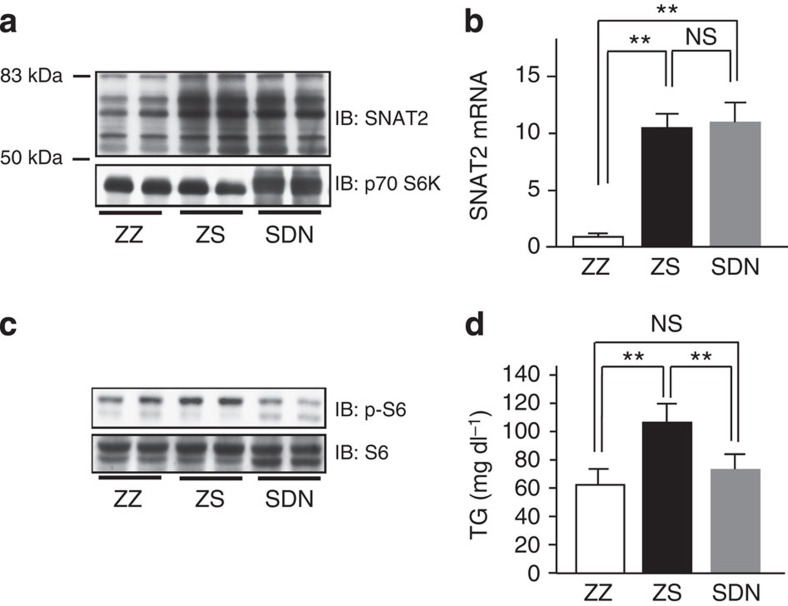
Blockage of SNAT2-induced hepatic mTORC1/S6K activation improves hypertriglyceridemia. (**a**–**d**) Mixed adenoviruses, that is, double-dose LacZ-adenovirus (ZZ), LacZ plus SNAT2 (ZS) and SNAT2 plus DN-S6K (SDN) adenoviruses, were administered to C57BL/6 mice. (**a**) Immunoblotting of liver extracts with anti-SNAT2 or p70-S6K antibodies. (**b**) SNAT2 mRNA expressions in the liver were analysed by RT–PCR (*n*=5–6). (**c**) Immunoblotting of liver extracts with anti-phospho-S6 or S6 antibodies. (**d**) Serum TG levels were measured on day 5 after adenovirus administration (*n*=4–6). The representative images derived from at least two experiments were displayed (**a** and **c**). Data are presented as means±s.d. **P<0.01 by the unpaired *t*-test. NS, not significant.

Next, to determine whether the hepatic mTORC1/S6K pathway is actually involved in the hypertriglyceridemia induced by hepatic SNAT2 expression, we blocked this pathway in SNAT2 mice by expressing the dominant-negative form of p70-S6kinase (DN-S6K)^32^ in the liver. Mixed recombinant adenoviruses, SNAT2 plus DN-S6K (SDN mice), LacZ plus SNAT2 (ZS mice) and double-dose LacZ (ZZ mice), were administered to C57BL/6 mice fed standard chow. Hepatic SNAT2 expression was similarly increased in ZS mice and SDN mice (Fig. 5a,b). Although S6 phosphorylation was enhanced in ZS mice, it was markedly suppressed in SDN mice (Fig. 5c). Under these conditions, SNAT2-induced hypertriglyceridemia was markedly inhibited by DN-S6K expression (Fig. 5d). Thus, hepatic mTORC1/S6K activation is responsible for alterations in systemic lipid metabolism induced by increased AA entry into the liver.

### A neuronal relay mediates the hypertriglyceridemia

**Figure 6:**
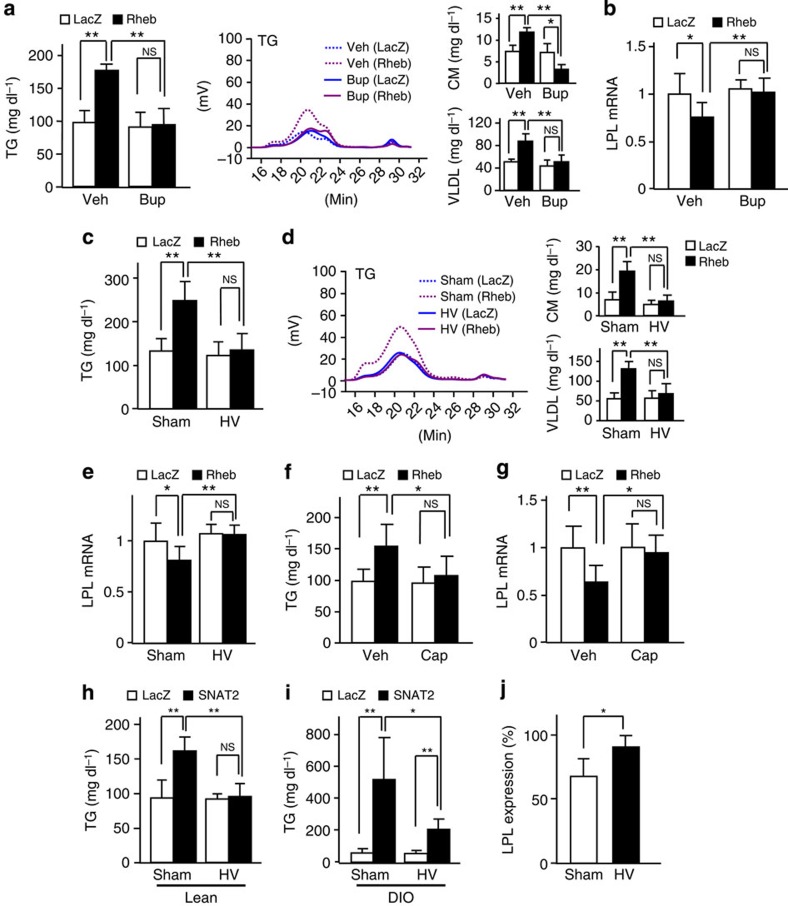
Hepatic AA/mTORC1/S6K activation raises serum TGs via a neuronal relay. Rheb (black bars) (**a**–**g**) or SNAT2 (black bars) (**h**–**j**) and control LacZ (white bars), adenoviruses were administered. (**a** and **b**) Vehicle (Veh) or bupranolol (Bup) was intraperitoneally administered to standard chow-fed C57BL/6 mice 5 days after adenoviral administration. Serum TG levels and HPLC results of sera in fed states were measured (**a**), and LPL mRNA expression in WAT was examined (**b**) 60 min after this administration (**a**; *n*=4–6, **b**; *n*=6–7). (**c**–**e**) Standard chow-fed C57BL/6 mice were subjected to sham operation (Sham) or HV 7 days before adenoviral administration. Serum TG levels in fed states were measured (**c**), sera in fed states were analysed by HPLC (**d**) and LPL mRNA expressions in WAT were determined (**e**) on day 5 after adenovirus administration (**c**; *n*=6–7, **d**; *n*=4–5, **e**; *n*=6–7). (**f**,**g**) Vehicle (Veh) or capsaicin (Cap) was applied to the hepatic vagus of standard chow-fed C57BL/6 mice 7 days before adenoviral administration. Serum TG levels in the fed state were measured (**f**) and LPL mRNA expression in WAT was examined (**g**) on day 5 after adenovirus administration (**f**; *n*=6–8, **g**; *n*=6–7). (**h**–**j**) The C57BL/6 mice were subjected to sham operation (Sham) or HV 7 days before adenoviral administration. Serum TG levels in fed states were measured (**h** and **i**), and the ratios of LPL mRNA expression in WAT, as compared with controls, were examined (**j**) on day 5 after adenovirus administration (**h**; *n*=4–5, **i**; *n*=4–5, **j**; *n*=5). Data are presented as means±s.d. *P<0.05, **P<0.01 by the unpaired *t*-test. NS, not significant.

Thus, hepatic AA/mTORC1/S6K activation induced hypertriglyceridemia involving adipose LPL downregulation. Next, we focused on this inter-tissue (liver to adipose) mechanism by which hepatic mTORC1/S6K activation suppresses LPL mRNA expression in WAT and elevates serum TG. LPL expression in WAT was reported to be negatively regulated by adrenergic stimulation^33^,^34^. Indeed, UCP1 and PGC1α mRNA expressions in WAT were upregulated in Rheb mice (Supplementary Fig. 8a), suggesting increased sympathetic outflow to WAT. Therefore, to examine whether sympathetic, especially β-adrenergic, activation mediates adipose LPL downregulation, we administered bupranolol, a β-adrenergic inhibitor, to Rheb mice. Bupranolol administration almost completely inhibited Rheb-induced elevation of serum TGs, especially in the chylomicron and VLDL fractions (Fig. 6a), as well as downregulation of LPL expression in WAT (Fig. 6b). Thus, the β-adrenergic function of the sympathetic nerve is involved in LPL downregulation in WAT and the resultant hypertriglyceridemia induced by hepatic mTORC1/S6K activation.

Therefore, the direct target of the signals from the liver may be the brain from which sympathetic signals originate, rather than WAT. Next, to address whether afferent neuronal signals from the liver are involved in regulating LPL expression in WAT, we performed selective hepatic vagotomy (HV). In Rheb mice fed standard chow, hepatic expression of Rheb (Supplementary Fig. 8b) and phosphorylation of p70-S6K (Supplementary Fig. 8c) were unaffected by HV. Under these conditions, HV almost completely blocked serum TG elevation in Rheb mice (Fig. 6c), especially in the chylomicron and VLDL fractions (Fig. 6d). HV blocked the downregulation of LPL expression (Fig. 6e) as well as the upregulations of UCP1 and PGC1α expressions in WAT of Rheb mice (Supplementary Fig. 8d). These findings indicate that neuronal signals consisting of the hepatic vagus and sympathetic nerves are involved in serum TG elevation induced by hepatic mTORC1/S6K activation.

HV involves dissection of both afferent and efferent vagal branches innervating the liver. Therefore, to confirm the involvement of afferent vagal signals originating in the liver, we applied a specific afferent neurotoxin, capsaicin, to the hepatic branch of the vagus. As reported previously^3^,^35^, perivagal capsaicin treatment did not affect immunoreactivity for S100 proteins, but did decrease expression of calcitonin gene-related peptide, a sensory neuropeptide (Supplementary Fig. 8e), while immunoreactivity for calcitonin gene-related peptide in the splanchnic nerve was not altered by this experimental procedure (Supplementary Fig. 8f), indicating selective deafferentation of the hepatic vagus. Hepatic Rheb expression levels induced by adenoviral administration were not affected by perivagal capsaicin treatment (Supplementary Fig. 8g). Under these conditions, selective deafferentation of the hepatic vagus in Rheb mice inhibited Rheb-induced elevation of serum TGs (Fig. 6f) as well as downregulation of LPL expression in WAT (Fig. 6g). Collectively, these findings suggest that hepatic mTORC1/S6K activation suppresses LPL expression in WAT via a neuronal relay consisting of the afferent vagus from the liver and sympathetic nerves to WAT.

Next, to confirm that the neuronal signals delivered via the hepatic vagus are indeed involved in hypertriglyceridemia induced by hepatic SNAT2 expression, HV was performed in SNAT2 mice fed standard chow. HV completely blocked hypertriglyceridemia in these mice as well (Fig. 6h), indicating that the hepatic vagus mediates the inter-tissue coordination whereby hepatic AA signalling affects systemic lipid metabolism. On the other hand, HV-induced inhibition of serum TG elevation was partial in DIO mice (Fig. 6i). These findings suggest that increased hepatic TG production/secretion induced by hepatic SNAT2 expression is not mediated by the aforementioned neuronal signals, leading to a partial blockade by HV in the obese mice. Again, HV also inhibited downregulation of LPL expression in WAT of DIO-SNAT2 mice (Fig. 6j). Taken together, these results indicate that afferent neuronal signals from the liver, transmitted through the vagus, mediate this inter-tissue (liver to adipose) and inter-nutrient (AA to lipid) communication.

### Hepatic mTORC1/S6K blockade inhibits TG elevation in obesity

**Figure 7:**
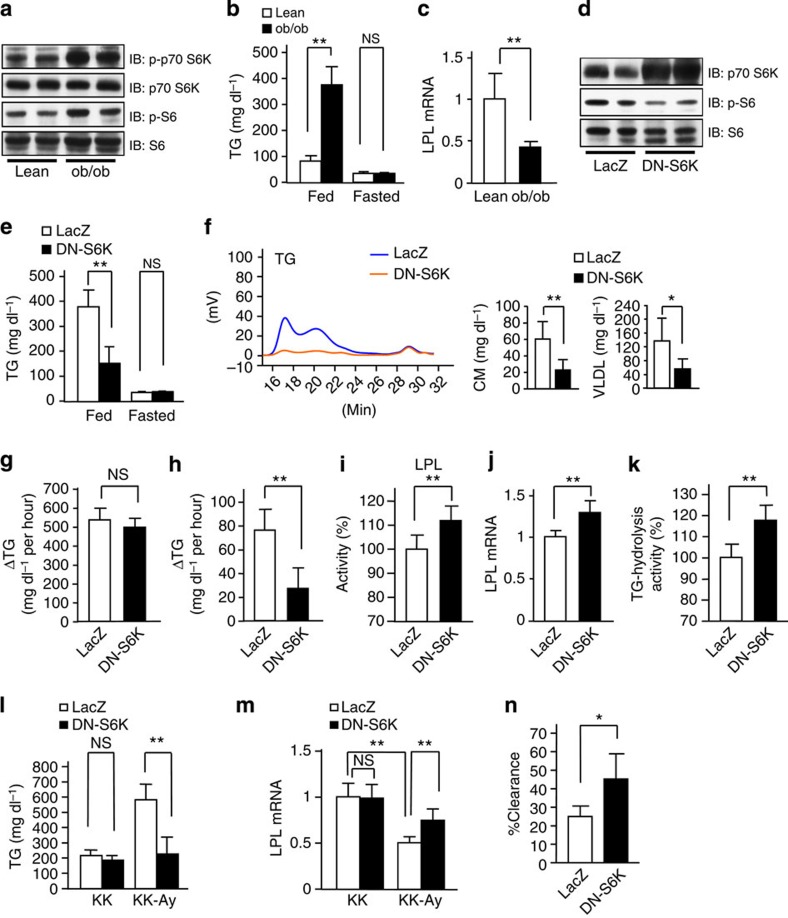
Inhibition of hepatic mTORC1/S6K pathway ameliorates obesity-related hypertriglyceridemia. (**a**) Immunoblotting of liver extracts from ob/ob mice and their lean control C57BL/6 mice with anti-phospho-p70-S6K, p70-S6K, phospho-S6 or S6 antibodies. (**b**) Serum TG levels were measured in ob/ob (black bars) and control (white bars) mice under 10 h-fasted or -fed conditions (*n*=5–7). (**c**) LPL mRNA expressions in WAT were examined (*n*=8–9). (**d**–**k**) DN-S6K (black bars) or LacZ (white bars) adenovirus was administered to ob/ob mice. (**d**) Liver extracts were immunoblotted with anti-p70-S6K, phospho-S6 or S6 antibodies. Serum TG levels were measured (**e**) and sera in fed states were analysed by HPLC (**f**) on day 5 after adenovirus administration (**e**; *n*=4–5, **f**; *n*=4–7). The rates of hepatic TG secretion were determined (**g**), and fat-tolerance tests were performed (**h**) (**g**; *n*=4–7, **h**; *n*=5). Plasma LPL activity (**i**), LPL mRNA expressions in WAT (**j**) and TG-hydrolysis activity in WAT (**k**) were determined on day 5 after adenovirus administration (**i**; *n*=6, **j**; *n*=7–11, **k**; *n*=6–7). (**l**–**n**) DN-S6K (black bars) or LacZ (white bars) adenovirus was administered to KK and KK-Ay mice. Serum TG levels (**l**) and LPL mRNA expression in WAT (**m**) were determined on day 5 after adenovirus administration (**l**; *n*=4–7, **m**; *n*=4–7). (**n**) TG-clearance studies were performed (*n*=5). The representative images derived from at least two experiments were displayed (**a** and **d**). Data are presented as means±s.d. *P<0.05, **P<0.01 by the unpaired *t*-test. NS, not significant.

Obesity is well known to be associated with hypertriglyceridemia, which is one of the major features of the metabolic syndrome. Impairment of LPL-mediated TG clearance is involved in the hypertriglyceridemia phenotype of the metabolic syndrome^28^. In addition, the hepatic mTORC1/S6K pathway is reportedly activated in obese states^15^,^16^. Therefore, we next examined whether this inter-tissue system is involved in obesity-related hypertriglyceridemia. In genetically obese ob/ob mice, hepatic AA concentrations were elevated (Fig. 1a), though hepatic SNAT2 expression showed compensatory downregulation (Supplementary Fig. 9). The mTORC1/S6K pathway was actually activated in the liver (Fig. 7a), and serum TGs were markedly elevated in the fed state (Fig. 7b). In association with the elevation of serum TGs in obesity, LPL expression in WAT was remarkably decreased in ob/ob mice compared with lean mice (Fig. 7c). To block the hepatic mTORC1/S6K pathway in this obesity model, we expressed DN-S6K using adenoviral gene transfer. Hepatic DN-S6K expression in ob/ob mice (DN-ob mice) reduced S6 phosphorylation in the liver (Fig. 7d), confirming suppression of hepatic S6K activity. Importantly, serum TGs in the fed state were significantly decreased by DN-S6K expression (Fig. 7e). Obesity-induced lipid profile abnormalities, including increased TG contents in the chylomicron and VLDL fractions, were markedly ameliorated in DN-ob mice (Fig. 7f). Hepatic DN-S6K expression did not affect hepatic TG secretion rates (Fig. 7g), and hepatic expressions of several genes related to lipid metabolism were unaltered in DN-S6K mice (Supplementary Fig. 10a,b). On the other hand, hepatic DN-S6K expression blunted the serum TG elevation after olive oil loading (Fig. 7h), suggesting enhanced TG degradation, that is, increased plasma TG hydrolysis. In fact, salt-inhibited TG-hydrolysis activities (by LPL) in the plasma were significantly increased (Fig. 7i) in association with upregulation (Fig. 7j) and increased activity (Fig. 7k) of LPL in WAT. In contrast to the obese state, in lean C57BL/6 mice, hepatic DN-S6K expression did not significantly affect serum TG concentrations (Supplementary Fig. 11a) or adipose LPL expression (Supplementary Fig. 11b).

We additionally examined the effects of hepatic S6K inhibition on obesity-related hypertriglyceridemia using another murine model, KK-Ay mice, with genetic obesity. KK-Ay mice also exhibited remarkable hypertriglyceridemia in the fed state (Fig. 7l) with decreased LPL expression in WAT (Fig. 7m). Hepatic DN-S6K expression markedly ameliorated hypertriglyceridemia in KK-Ay mice (Fig. 7l) with increased adipose LPL expression (Fig. 7m) and increased serum TG clearance after Intralipid injection (Fig. 7n). On the other hand, hepatic DN-S6K expression did not affect serum TGs in lean KK mice (Fig. 7l) with unaltering adipose LPL expression (Fig. 7m). The comparison between KK and KK-Ay mice indicates that suppression of the hepatic mTORC1/S6K pathway in KK-Ay mice restored obesity-related adipose LPL downregulation and serum TG elevation (Fig. 7l,m) to degrees similar to those in lean KK mice (Fig. 7l,m). Taken together, these results indicate that hepatic mTORC1/S6K signalling is activated under conditions of obesity and that this activation plays an important role in obesity-related hypertriglyceridemia.

### Increased LPL activity inhibits TG elevation in obesity

**Figure 8:**
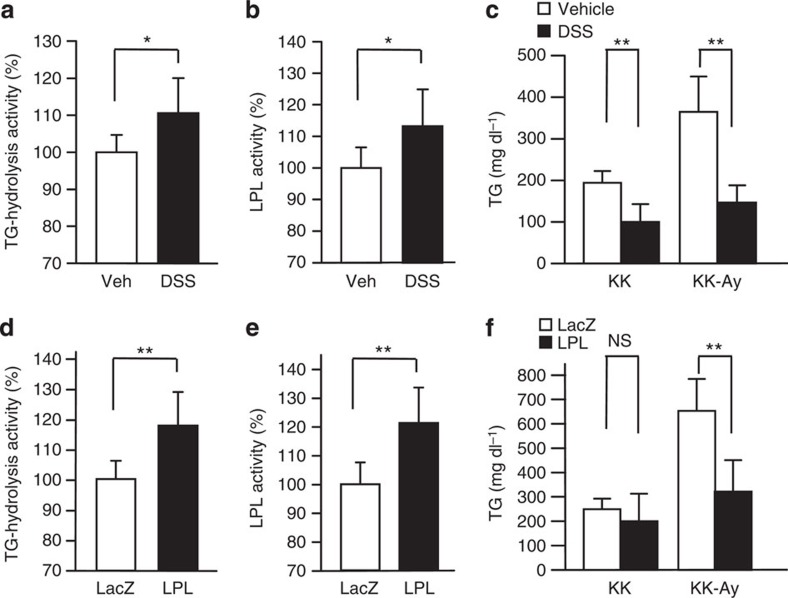
Upregulation of plasma TG-hydrolysis activities relieves obesity-related hypertriglyceridemia. (**a**–**c**) Vehicle (Veh) or dextran sodium sulfate (DSS) was intravenously administered to standard chow-fed KK and KK-Ay mice. Plasma TG-hydrolysis activities (**a**) and LPL activities (**b**) were measured, and serum TG levels (**c**) were examined 30 min after the injection of these agents (**a**; *n*=7–8, **b**; *n*=7–8, **c**; *n*=5–7). (**d**–**f**) LPL (black bars) or LacZ (white bars) adenovirus was administered to KK and KK-Ay mice. Plasma TG-hydrolysis activities (**d**) and LPL activities (**e**) were measured, and serum TG levels (**f**) were examined 5 days after adenoviral administration (**d**; *n*=5–8, **e**; *n*=5–8, **f**; *n*=6–8). Data are presented as means±s.d. *P<0.05, **P<0.01 by the unpaired *t*-test. NS, not significant.

Next, to examine whether increased plasma TG-hydrolysis activity improves obesity-related hypertriglyceridemia, we administered either DSS or LPL adenovirus to KK-Ay mice. First, DSS administration increased plasma TG-hydrolysis activity (Fig. 8a) and salt-inhibited TG-hydrolysis activity, that is, LPL activity, by 10–15%, compared with vehicle-treated mice (Fig. 8b), to an extent similar to those in obese DN-S6K mice (Fig. 7i), leading to improvement of obesity-related hypertriglyceridemia (Fig. 8c). Second, we adenovirally expressed LPL in obese KK-Ay mice. Plasma TG-hydrolysis activity (Fig. 8d) and LPL activity (Fig. 8e) were increased, by almost 15% as compared with LacZ mice, to an extent similar to those in obese DN-S6K mice (Fig. 7i), resulting in the improvement of obesity-related hypertriglyceridemia (Fig. 8f). These results confirmed that alterations in plasma TG-hydrolysis (LPL) activity are involved in the mechanism underlying obesity-related hypertriglyceridemia.

### Hepatic vagus contributes to hypertriglyceridemia in obesity

**Figure 9:**
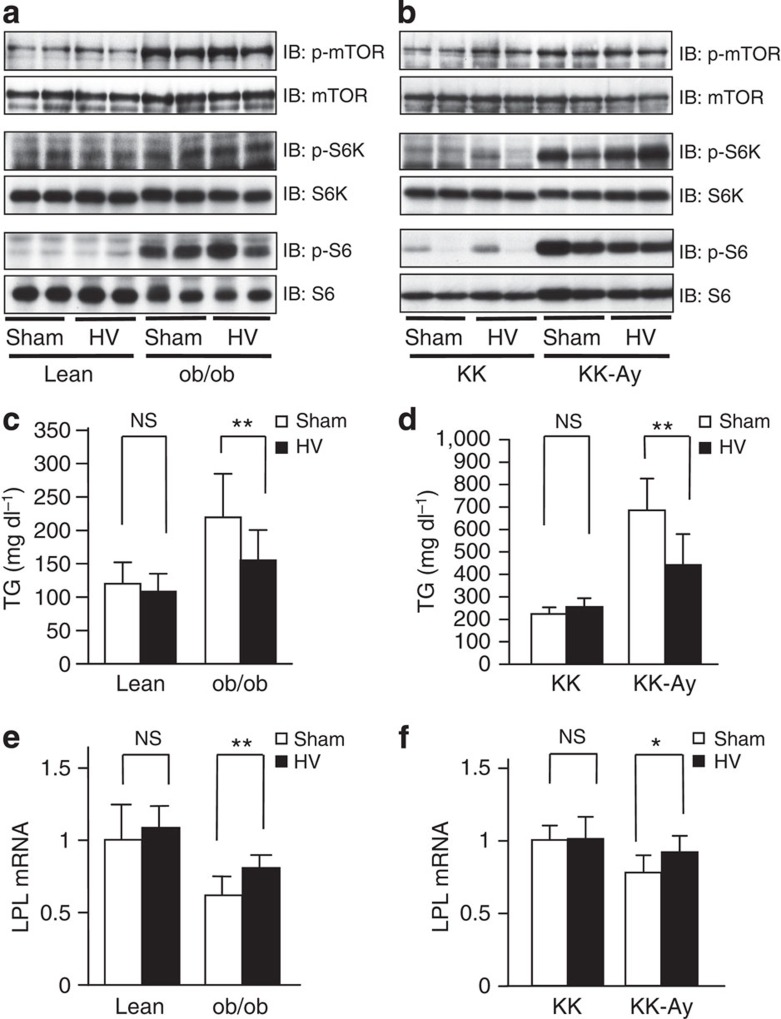
Blockage of hepatic vagus suppresses the development of hypertriglyceridemia associated with obesity. (**a**,**c**,**e**) Standard chow-fed C57BL/6 and ob/ob mice were subjected to sham operation (Sham) or HV. (**a**) Immunoblotting of liver extracts from these sham- and HV-mice with anti-phospho-mTOR, mTOR, phospho-p70-S6K, p70-S6K, phospho-S6 and S6 antibodies. Serum TG levels (**c**) and LPL mRNA expressions in WAT (**e**) were examined on day 14 after these operations (**c**; *n*=15–19, **e**; *n*=7–9). (**b**,**d**,**f**) Standard chow-fed KK and KK-Ay mice were subjected to sham operation (Sham) or HV. (**b**) Immunoblotting of liver extracts from these sham- and HV-mice with anti-phospho-mTOR, mTOR, phospho-p70-S6K, p70-S6K, phospho-S6 and S6 antibodies. Serum TG levels (**d**) and LPL mRNA expressions in WAT (**f**) were examined on day 14 after these operations (**d**; *n*=6–7, **f**; *n*=5–9). The representative images derived from at least two experiments were displayed (**a** and **b**). Data are presented as means±s.d. *P<0.05, **P<0.01 by the unpaired *t*-test. NS, not significant.

Next, we aimed to investigate whether signals mediated by the hepatic vagus are actually involved in the process of dysregulation of systemic lipid metabolism during obesity development. We performed selective HV or sham operation on genetically obese (ob/ob and KK-Ay) mice and their lean counterparts (C57BL/6 and KK, respectively) at 7 weeks of age, followed by the measurement of serum TG levels. Selective HV did not affect endogenous mTOR and S6kinase phosphorylation in the livers of either lean or obese mice (Fig. 9a,b). HV alone suppressed serum TG elevation associated with obesity development, in both ob/ob (Fig. 9c) and KK-Ay (Fig. 9d) mice. In contrast, HV did not affect serum TG levels in their lean counterparts (Fig. 9c,d). Thus, selective HV protected ob/ob and KK-Ay mice from the progression of hypertriglyceridemia associated with obesity. Furthermore, adipose LPL expressions were decreased in sham-operated obese mice, but HV reversed the decreased adipose LPL expressions associated with obesity development, in both ob/ob (Fig. 9e) and KK-Ay (Fig. 9f) mice. In contrast, HV did not affect hepatic expressions of molecules related to hepatic lipid metabolism, including MTP, LDLR, SREBP1c and PPARα, in either ob/ob (Supplementary Fig. 12a), KK-Ay (Supplementary Fig. 12b) or their lean counterparts (Supplementary Fig. 12a,b). HV, likewise, did not affect hepatic expressions of secreted proteins related to plasma TG hydrolysis, including apoC-2 and -3, angptl-3, -4 and -8, and GPIHBP1 in ob/ob (Supplementary Fig. 12c), KK-Ay (Supplementary Fig. 12d) or their lean counterparts (Supplementary Fig. 12c,d). These results support the pathophysiological significance of the concept that signals from the hepatic vagus, which functions to regulate adipose LPL via a neuronal pathway, can contribute to the derangement of systemic lipid metabolism associated with the development of obesity.

## Discussion

**Figure 10:**
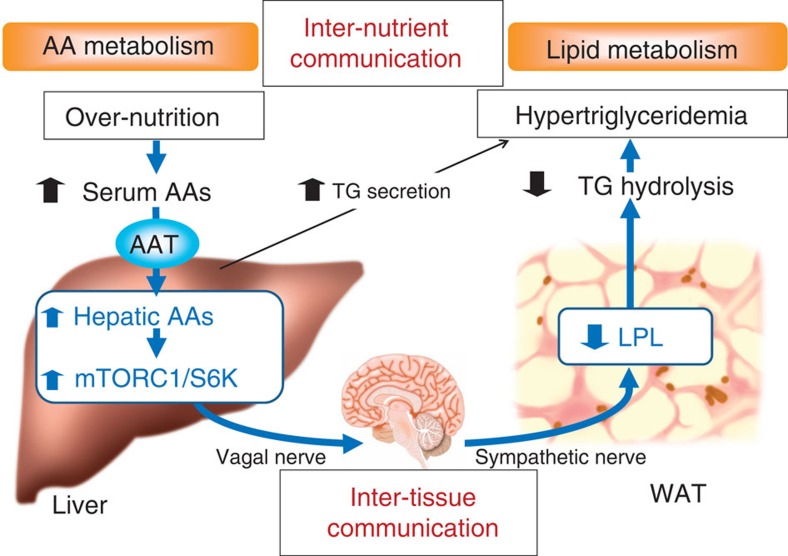
Scheme of the pathophysiology of obesity-related hypertriglyceridemia. Hepatic AA signalling regulates systemic lipid metabolism via a neuronal pathway. This neuronal relay from the liver to adipose tissue is mediated by the hepatic mTORC1/S6K pathway. This neuronal inter-tissue (liver to adipose) system decreases adipose LPL expression and suppresses plasma TG-hydrolysis activity, thereby raising serum TG levels. Namely, this neuronal mechanism produces inter-nutrient (AA to lipid) coordination. Furthermore, excessive activation of this pathway, under conditions of sustained over-nutrition during obesity development, contributes to obesity-related hypertriglyceridemia.

In this study, our aim was to elucidate the roles of hepatic AA entry and the resultant mTOR/S6K activation in systemic metabolism. As a tool for increasing AA influx into the liver, we expressed an AAT, SNAT2. In fact, adenoviral SNAT2 expression in the liver successfully increased AA concentrations and mTORC1/S6K activation in the liver. Not only SNAT2 but also Rheb expression, which activates the mTORC1/S6K pathway in the liver, induced hypertriglyceridemia, especially in postprandial states, via decreased TG hydrolysis mainly owing to adipose LPL downregulation. Pharmacological LPL activation as well as adenoviral LPL expression actually reversed Rheb-induced decreases in plasma TG-hydrolysis activity and inhibited Rheb-induced serum TG elevations. Furthermore, LPL inhibition induced by either lipase inhibitor administration or adenoviral angptl-4 expression similarly raised serum TG levels. Thus, these gain-of-function and loss-of-function experiments revealed the involvement of LPL in the mechanism of hypertriglyceridemia observed in Rheb mice. DN-S6K expression blocked SNAT2-induced elevation of serum TG levels, indicating the hepatic AA/mTORC1/S6K pathway to underlie this mechanism. In addition, denervation, pharmacological deafferentation and β-blocker administration revealed that a neuronal relay, consisting of afferent vagal and efferent sympathetic nerves, mediates this liver-to-adipose metabolic coordination. Furthermore, inhibition of hepatic mTORC1/S6K pathway and blockage of hepatic vagus in the states of obesity revealed that this neuronal relay system has a contribution to the progression of obesity-related hypertriglyceridemia. Namely, this inter-tissue mechanism produces inter-nutrient coordination between AAs and lipids at the whole-body level (Fig. 10).

Association of the mTORC1/S6K pathway with lipid metabolism has recently been the focus of research on fatty acid biosynthesis^36^,^37^,^38^ and autophagy-regulated lipid metabolism^39^. The mTORC1/S6K pathway has been shown to regulate expression of lipogenesis- and glycolysis-related genes *in vitr*o^40^ and manipulation of this pathway reportedly modulates lipogenesis *in vivo*^41^,^42^. mTORC1 is required for postprandial *de novo* lipogenesis in the murine liver^43^. Thus, the mTORC1/S6K pathway is considered to function as an energy sensor, thereby modulating cellular processes involved in lipid metabolism^44^. In addition to these intracellular regulations, the present study elucidated a novel inter-tissue mechanism which regulates systemic lipid metabolism originating in the mTORC1/S6K pathway in the liver. Given that the serum TG elevations in both SNAT2 and Rheb mice fed standard chow were almost completely inhibited by selective blockade of either the hepatic vagus or the β-adrenergic sympathetic nerve, this neuronal relay originating in hepatic AA/mTORC1/S6K signalling presumably plays a major role in the inter-nutrient metabolic regulation between AAs and lipids.

Consistent with this notion, intraportal AA infusion reportedly stimulates both the afferent hepatic vagus^45^ and sympathetic nerves^46^. Neuronal signals have recently attracted increasing attention for their roles in the metabolic network system. In particular, the hepatic vagus has recently been revealed to transmit a variety of metabolic information, including enhanced lipid storage^3^ and glucose metabolism^5^, leading to different metabolic alterations in other tissues. The present study identified a novel mechanism whereby afferent neuronal signals from the liver, originating in hepatic AA metabolism affect systemic lipid metabolism. Thus, metabolic information regarding the three major nutrients may be dynamically exchanged via the autonomic nervous system at the whole-body level. The liver may be the origin of information about nutrient metabolism which changes from second to second. The nutrients obtained from food are delivered directly to the liver via the portal vein, indicating that the liver is situated in an optimal position for receiving and sending nutrient information and that a variety of information is transmitted via the afferent hepatic vagus. In addition, interestingly, neuronal blockades, such as those achieved by bupranolol administration, hepatic vagotomy and pharmacological deafferentation, did not alter the serum TG levels in control mice, though TG elevations induced by hepatic Rheb expression or in obesity models were markedly inhibited by each form of neuronal blockade. These findings demonstrate that neuronal relay signals exert minimal effects in lean states, while strongly functioning under conditions of hepatic AA/mTOR/S6K activation, for example, obesity.

Another interesting finding in this study is that blockade of this mechanism lowered serum TG levels in both ob/ob and KK-Ay mice, indicating that the inter-tissue system contributes to the development of obesity-related hypertriglyceridemia. Hypertriglyceridemia is a major feature of the metabolic syndrome and is an important cause of cardiovascular morbidity^47^,^48^. It is well known that impaired LPL-mediated TG clearance in addition to increased hepatic VLDL secretion explains the hypertriglyceridemia phenotype of the metabolic syndrome^28^. In murine models, plasma TG-hydrolysis activity detected in post-heparin plasma showed an inverse relation to serum TG levels^49^. In human subjects as well as murine models, slight decreases in plasma TG-hydrolysis activity, by ∼10%, are reportedly associated with serum TG elevation^50^,^51^,^52^. In the present study, either DSS administration or adenoviral LPL expression which similarly increased plasma TG-hydrolysis activity in obese KK-Ay mice, markedly improved hypertriglyceridemia. Taken together with the observation that hepatic DN-S6K expression in the livers of both ob/ob and KK-Ay mice inhibited hypertriglyceridemia, these findings strongly suggest that the decrease in TG-hydrolysis activity induced by the inter-tissue mechanism contributes to pathological conditions in the metabolic syndrome.

There are several sources of TG-hydrolysis activity^27^,^28^. A study using cardiac-specific LPL-deficient mice revealed the importance of cardiac LPL in regulating serum TG levels^53^. However, in SNAT2 mice, LPL expression as well as LPL activity in WAT alone, among all the examined tissues, was downregulated. We cannot rule out the possibility that another unknown enzyme involved in TG-hydrolysis activity is responsible for the hypertriglyceridemia observed in SNAT2 mice. However, salt-inhibited, but not salt-insensitive, TG-hydrolysis activities were decreased in SNAT2 mice, indicating decreased activity of LPL. In addition, hepatic SNAT2 and Rheb expressions downregulated adipose LPL expression and raised serum TG levels. Blockade of the hepatic S6K pathway, hepatic vagal signals or the β-adrenergic system reversed adipose LPL downregulation and lowered serum TG levels. Furthermore, adipose LPL expressions are known to be under negative regulation by adrenergic stimulation *in vitro*^54^,^55^ and *in vivo*^33^,^34^. These findings suggest that adipose LPL downregulation plays an important role in decreased TG-hydrolysis activity and the resultant hypertriglyceridemia in SNAT2 and Rheb mice.

What then is the physiological role of this inter-tissue and inter-nutrient mechanism? Among nutrients, AAs, which activate the mTORC1/S6K pathway, are well known to be utilized for nutrition sensing. For instance, AA deficiency intracellularly induces autophagy via alterations in the mTORC1/S6K pathway to make up for nutrient deficiencies^56^,^57^. On the other hand, AA excess may represent states of over-nutrition. As the liver efficiently receives the nutritional information derived from foods via the portal vein, it can function as a metabolic sensor tissue at the whole-body level. In fact, high-protein diet loading, that is, surplus intake of dietary AAs, reportedly enhances postprandial TG elevation in both mice^58^,^59^ and humans^60^. The present results indicate that, in the state of obesity, the hepatic mTORC1/S6K pathway is physiologically activated, leading to downregulation of LPL in adipose tissue via a neuronal relay. LPL suppression decreases lipid uptake and utilization in peripheral tissues by inhibiting TG breakdown into fatty acids. Therefore, it is considered that this mechanism leads to systemic negative feedback responses to over-nutrition. However, this inter-nutrient mechanism induces marked hypertriglyceridemia, one of the major features of the metabolic syndrome in the state of obesity.

As for glucose metabolism, it is well known that obesity induces insulin resistance, thereby suppressing glucose transport and utilization in peripheral tissues^61^. Insulin resistance is considered to play a physiological role in preventing further energy accumulation and obesity, but it induces diabetes and many other disorders of the metabolic syndrome under conditions of sustained over-nutrition. Therefore, these two mechanisms, that is, insulin resistance and the neuronal relay identified in this study, might be conceptually analogous in terms of glucose and lipid metabolism, respectively. These mechanisms might have worked advantageously when foods were repeatedly in excess or short supply, but, in the current age of constant plenty, they ironically trigger obesity-related disorders. On the basis of several clinical studies, hypertriglyceridemia is a well-known independent risk for atherosclerosis development^47^,^48^. The hepatic mTORC1/S6K pathway and neuronal signals may constitute a potential therapeutic target for the prevention of obesity-related hypertriglyceridemia.

## Methods

### Animals

Animal studies were conducted in accordance with Tohoku University institutional guidelines. All the experimental protocols have been approved by the Institutional Animal Care and Use Committee of the Tohoku University Environmental & Safety Committee. Male C57BL/6N mice, KK mice and KK-Ay mice were purchased from Japan Clea (Tokyo, Japan). Male ob/ob mice were purchased from Japan Charles River Laboratory (Yokohama, Japan). DIO mice were obtained by 4-week feeding of a high-fat diet (32% safflower oil, 33.1% casein, 17.6% sucrose and 5.6% cellulose^3^) beginning at 5 weeks of age.

### Preparation of recombinant adenoviruses

Murine SNAT2 (slc38a2) cDNA and murine angptl-4 cDNA were obtained by reverse transcriptase-polymerase chain reaction (RT–PCR) with total RNA obtained from murine livers. Recombinant adenovirus, containing the murine SNAT2 and angptl-4 under the control of the CMV promoter, was prepared as described previously^3^. Recombinant adenovirus containing the bacterial β-galactosidase gene^21^ was used as a control. Recombinant adenoviruses containing murine Rheb, the constitutively active form of p70-S6Kinase, and the dominant negative form of p70-S6Kinase1 were used as reported previously^32^. Recombinant adenovirus, containing the murine LPL under the control of the CMV promoter, was generated using the Adeno-X Adenoviral System 3 (Takara Bio Inc., Otsu, Japan).

### Administration of recombinant adenoviruses

Recombinant adenoviruses bearing murine SNAT2 (slc38a2) cDNA or the bacterial β-galactosidase gene were injected into the tail veins of standard chow-fed and DIO male C57BL/6N mice at a dose of 1.0 × 10^8^ plaque-forming units. Recombinant adenoviruses bearing Rheb cDNA or the constitutively active form of p70-S6Kinase were also injected into the tail veins of standard chow-fed male C57BL/6N mice at a dose of 1.0 × 10^8^ plaque-forming units. Recombinant adenovirus-bearing angptl-4 cDNA was also injected into the tail veins of standard chow-fed male C57BL/6N mice at a dose of 6.6 × 10^7^ plaque-forming units. Recombinant adenovirus bearing the dominant-negative form of the p70-S6kinase1 gene was injected into the tail veins of 7-week-old male C57BL/6N, ob/ob, KK and KK-Ay mice fed standard chow at a dose of 1.0 × 10^8^ plaque-forming units. Recombinant adenovirus bearing LPL cDNA was injected into the tail veins of 7-week-old male C57BL/6N, KK and KK-Ay mice fed standard chow at a dose of 1.0 × 10^8^ plaque-forming units.

### Immunoblotting

Tissue samples were homogenized in ice-cold lysis buffer containing 100 mM Tris, pH 8.5, 250 mM NaCl, 1% Nonidet P-40, 1 mM EDTA, 1 mM phenylmethylsulfonyl fluoride, aprotinin at 1:5,000 dilution and leupeptin at 1:5,000 dilution, as previously described^3^. Tissue homogenates were centrifuged and the supernatants including tissue protein extracts (250 μg total protein) were boiled in Laemmli buffer containing 10 mM dithiothreitol, then subjected to SDS–polyacrylamide gel electrophoresis (SDS–PAGE). The liver lysates were immunoblotted with antibodies including anti-SNAT2 antibody (sc-67081), anti-angptl-4 antibody (sc-66807; Santa Cruz Biotechnology, Inc., Santa Cruz, CA, USA), anti-Rheb antibody (#4935), anti-mTOR antibody (#2972), anti-phospho-mTOR (Ser2448) antibody (#2971), anti-p70-S6Kinase antibody (#2708), anti-phospho-p70-S6Kinase (Thr389) antibody (#9234), anti-S6 ribosomal protein antibody (#2217) and anti-phospho-S6 ribosomal protein (Ser235/236) antibody (#4857) (Cell Signaling Technology, Danvers, MA, USA). The lysates from WAT were immunoblotted with anti-LPL antibody (sc-32885; Santa Cruz Biotechnology, Inc.). The immunoblots using antibodies provided from Santa Cruz or Cell Signaling Technology were performed typically at 1:600 or 1:3,000 dilution in Can Get Signal (TOYOBO, Osaka, Japan), respectively, followed by secondary antibodies (HRP conjugated) at 1:10,000 dilution. The immunoblots were visualized with an enhanced chemiluminescence detection kit (Amersham, Buckinghamshire, UK). In particular, endogenous or overexpressed SNAT2 protein had several bands in blots encompassing a broad range^62^. This is considered to result from SNAT2 protein being glycosylated^63^ and ubiquitinated^64^ in different ways. Uncropped scans of western blotting are provided in Supplementary Figs 13–19.

### Immunoprecipitation

Mice fasted 16 h were injected with 200 μl of normal saline, with or without 10 U kg^−1^ body weight of insulin, via the tail vein. The liver was removed 300 s later and was homogenized in ice-cold lysis buffer containing 50 mM Tris, pH 7.4, 100 mM NaCl, 10 mM EDTA, 10% glycerol, 1% Nonidet P-40, 2 mM sodium orthovanadate, 1 mM phenylmethylsulfonyl fluoride, 40 mM β-glycerophosphate and 50 mM NaF. The tissue homogenates were centrifuged and the supernatants including tissue protein were used for immunoprecipitation with 5 μl of anti-Akt antibody (#9272; Cell Signaling Technology), coupled with protein A-Sepharose (GE Healthcare, Buckinghamshire, UK). Next, the immunoprecipitates were subjected to SDS–PAGE and then immunoblotted using anti-phospho Akt (Ser473) antibody (#9271) or anti-Akt antibody (Cell Signaling Technology) at 1:3,000 dilution in Can Get Signal (TOYOBO), followed by secondary antibodies (HRP conjugated) at 1:10,000 dilution, as described previously^3^. Uncropped scans of western blotting are provided in Supplementary Fig. 19.

### Blood analysis

Serum TG levels were determined with a Lipidos liquid kit (TOYOBO, Osaka, Japan). Plasma TG-hydrolysis activity was measured with an LPL activity kit (Roar Biomedical, Inc., New York, NY, USA) according to the manufacturer's instructions with slight modifications. Serum AA concentrations were measured commercially (SRL, Tokyo, Japan). Serum lipoproteins were analysed by high-performance liquid chromatography (HPLC) using molecular sieve columns (Skylight Biotech, Akita, Japan).

### Measurement of lipid profiles in serum lipoproteins

The lipid profiles of serum lipoproteins were analysed using a dual detection HPLC system with two tandem connected TSKgel LipopropakXL columns (300 mm × 7.8 mm; Tosoh, Tokyo, Japan) according to the methods^65^ established by Skylight Biotech (Akita, Japan). For HPLC analysis, the graphs show data from one mouse, representing each of the two or four groups studied. Similar results were obtained with other mice (*n*=4–5) from each experimental group.

### Measurement of plasma TG-hydrolysis activity

Mice, under 4 h-fasted conditions, were injected intravenously with 300 IU heparin per kilogram body weight. Blood samples were obtained 10 min after heparin infusion. Heparinized plasma was prepared for the determination of TG-hydrolysis activity using the LPL activity kit (Roar Biochemical, Inc.) according to the manufacturer's instructions with slight modifications^66^,^67^. The NaCl concentration of the assay buffer normally used according to the manufacturer's instructions was 150 mM. For a high-salt condition, the NaCl concentration of the assay buffer was 1 M. This assay includes a nonfluorescent substrate emulsion, which becomes intensely fluorescent upon interaction with LPL. Heparinized plasma was diluted 10-fold by either normal or high-salt assay buffers, and 10 μl of the diluted plasma were mixed in a 96-well microplate with 200 μl of substrate emulsion. After incubation at room temperature for 15 min, the fluorescent intensity of these reaction mixtures obtained using these buffers was measured with a fluorescence microplate reader (LabSystems, Kilsyth, Victoria, Australia). In addition, pre-hydrolysed standard substrates at concentrations ranging from 0.001 to 2.0 μmol ml^−1^ were used to construct standard curves under normal and high-salt conditions. Finally, the relative amounts of TG-hydrolysis activity in plasma under normal and high-salt conditions were calculated from the respective standard curves. Plasma HL activity was obtained as the salt-insensitive TG lipase activity under the high-salt procedure. Plasma LPL activity, the salt-inhibited TG lipase activity, was obtained by subtracting plasma HL activity from plasma TG-hydrolysis activity measured using the routine procedure.

### Hepatic AA concentrations

The samples were prepared as reported previously with slight modifications^22^. Frozen livers were homogenized and centrifuged, and protein concentrations of the supernatants were determined by the BCA method. The samples were filtered through a 0.22 μm MILLEX filter (Millipore Co., Billerica, MA, USA) to remove precipitated proteins and tissue debris. Free AA concentrations of the sample were analysed commercially (SRL).

### Hepatic TG contents

Frozen livers were homogenized and TGs were extracted with CHCl_3_: CH_3_OH (2:1, v-v), dried and resuspended in 2-propanol^3^. TG contents were measured using Lipidos liquid (TOYOBO).

### TG secretion rate

To assess hepatic TG secretion, 500 mg kg^−1^ Triton WR-1339 (Tyloxapol, Sigma-Aldrich Co., St Louis, MO, USA), which blocks lipolysis of TGs in peripheral tissue, was injected into the tail veins of 6 h-fasted mice 5 days after adenovirus administration. Blood samples were taken immediately before and 30, 60 and 90 min after injection, followed by measurement of serum TG concentrations.

### Measurement of TG-hydrolysis activities in various tissues

The samples were homogenized with the elution buffer containing 100 mM Tris, 150 mM NaCl, 4 mM KCl, 3 mM CaCl_2_·2H_2_O, 2 mM MgSO_4_·7H_2_O, 10 g l^−1^ bovine serum albumin (Sigma-Aldrich Co.) and 10,000 IU l^−1^ heparin, with HCl adjusted to a pH of 7.4 (ref. 68). The homogenized samples were sonicated and incubated for 1 h at room temperature with gentle shaking. After centrifugation, the supernatants were subjected to determination of both the fluorescent intensity and protein concentrations using the LPL activity kit (Roar Biochemical, Inc.) and the BCA method (Thermo Fisher Scientific Inc., Wilmington, DE, USA), respectively. The relative amounts of TG-hydrolysis activity in these supernatants were calculated from standard curves obtained by pre-hydrolysed substrates. Finally, the relative amounts of TG-hydrolysis activities in various tissues were calculated per protein concentrations.

### Fat-tolerance tests

Fat-tolerance tests were performed on overnight-fasted (12 h) mice 5 days after adenovirus administration. These mice received gastric gavage of olive oil (20μl g^−1^ of body weight, Sigma-Aldrich Co.), followed by measurement of serum TG concentrations^23^ or HPLC analysis.

### TG-clearance studies

To assess the clearance of serum TG, Intralipid (100 μl of a 2.6% solution in sterile saline, Sigma-Aldrich Co.) was injected into the tail veins of mice 5 days after adenovirus administration. Blood samples were taken at 2 and 30 min after injection, followed by measurement of serum TG concentrations. Serum TG reportedly peaked 2 min after Intralipid injection^69^. Then, the decreases in serum TG at 30 min from the peak were demonstrated as % clearance 30 min after Intralipid injection.

### Dextran sodium sulfate (DSS) administration test

DSS (20 mg kg^−1^ of body weight, Sigma-Aldrich Co.), an LPL activator^70^, was intravenously administered to Rheb-, KK and KK-Ay mice. Blood samples were obtained at 30 min after DSS administration, followed by TG measurements. For measurement of plasma TG-hydrolysis activities, these mice were injected intravenously with 300 IU heparin per kilogram of body weight at 30 min after DSS administration, and heparinized plasma samples were obtained 10 min after heparin infusion.

### Dissection of hepatic branch of the vagus

Selective HV was performed as previously described^3^,^35^. A laparotomy incision was made on the ventral midline and the abdominal muscle wall was opened with a second incision. The gastrohepatic ligament was severed using fine forceps, and the stomach was gently retracted, revealing the descending ventral oesophagus and the ventral subdiaphragmatic vagal trunk. The hepatic branch of this vagal trunk was then transected using fine forceps.

### Selective hepatic vagal afferent blockade by perivagal application of capsaicin

Eight-week-old C57BL/6N mice were subjected to selective perivagal application of capsaicin as previously described^3^,^35^. The hepatic branch of the vagal trunk was exposed as described above, isolated from surrounding tissues using paraffin films, then loosely tied with a cotton string with or without being immersed in capsaicin (Sigma-Aldrich Co.) dissolved in olive oil (5% wt/vol). The cotton string was removed 30 min later and the abdominal incision was closed.

### Administration of a pan β-adrenergic inhibitor, bupranolol

A pan β-adrenergic inhibitor, bupranolol (30 mg kg^−1^ of body weight, Kaken Pharmaceutical Co., Ltd. Tokyo, Japan) was intraperitoneally administered to Rheb mice. Blood samples were obtained at 60 min after bupranolol administration, followed by TG measurements and lipoprotein HPLC analyses.

### Histological analysis

For hepatic vagal nerve immunohistochemistry, the oesophagus and vagal trunks were removed and fixed with 10% formalin and embedded in paraffin. In addition, splanchnic nerve was removed and fixed with 10% formalin and embedded in paraffin. Tissue sections were stained with haematoxylin–eosin. The streptavidin–biotin method was performed using a Histofine SAB-PO kit (Nichirei, Tokyo, Japan) and antibodies against calcitonin gene-related peptide (AB5920), tyrosine hydroxylase (AB152; Chemicon International, Temecula, CA, USA) and S-100 protein (Z0311; DAKO, Glostrup, Denmark). The antigen–antibody complex was visualized with 3,3'-diaminobenzidine and counterstained with haematoxylin^3^,^4^,^35^.

### Quantitative RT–PCR-based gene expression

Total RNA was isolated from 50 mg of mouse liver, epididymal WAT, brown adipose tissue, cardiac and skeletal muscles with Isogen (Wako Pure Chemical), and cDNA was synthesized with a Transcriptor First Strand cDNA Synthesis Kit (Roche Diagnostics Gmbh, Mannheim, Germany) using 5 μg of total RNA. cDNA synthesized from total RNA was evaluated with a real-time PCR quantitative system^3^ (Light Cycler Quick System 350S; Roche Diagnostics Gmbh). The relative amount of mRNA was calculated with 28SrRNA or β-actin mRNA as the invariant control. The primers used are described in the Supplementary Table 2.

### Orlistat administration test

Orlistat (Sigma-Aldrich Co.) was solubilized in 33% ethanol and 66% PEG 400 (Hampton Research, Aliso Viejo, CA, USA). The C57BL/6 mice fed a standard chow diet received orlistat (250 mg kg^−1^ body weight) via injection into the intraperitoneal space^26^, followed 90 min later by measurement of plasma TG-hydrolysis activities. Next, serum TG levels were measured and HPLC analyses of lipid profiles were performed.

### Statistical analysis

All the data are expressed as means±s.d. The statistical significance of differences was assessed by the unpaired *t-*test.

## Additional information

**How to cite this article:** Uno, K. *et al.* A hepatic amino acid/mTOR/S6K-dependent signalling pathway modulates systemic lipid metabolism via neuronal signals. *Nat. Commun.* 6:7940 doi: 10.1038/ncomms8940 (2015).

## Supplementary Material

Supplementary Information Supplementary Figures 1-19 and Supplementary Tables 1-2

## References

[b1] Katagiri H., Yamada T. & Oka Y. Adiposity and cardiovascular disorders: disturbance of the regulatory system consisting of humoral and neuronal signals. Circ. Res. 101, 27–39 (2007). 17615379 10.1161/CIRCRESAHA.107.151621

[b2] Muoio D. M. & Newgard C. B. Obesity-related derangements in metabolic regulation. Annu. Rev. Biochem. 75, 367–401 (2006). 16756496 10.1146/annurev.biochem.75.103004.142512

[b3] Uno K. *et al.* Neuronal pathway from the liver modulates energy expenditure and systemic insulin sensitivity. Science 312, 1656–1659 (2006). 16778057 10.1126/science.1126010

[b4] Imai J. *et al.* Regulation of pancreatic beta cell mass by neuronal signals from the liver. Science 322, 1250–1254 (2008). 19023081 10.1126/science.1163971

[b5] Tsukita S. *et al.* Hepatic glucokinase modulates obesity predisposition by regulating BAT thermogenesis via neural signals. Cell Metab. 16, 825–832 (2012). 23217261 10.1016/j.cmet.2012.11.006

[b6] Tremblay F., Lavigne C., Jacques H. & Marette A. Role of dietary proteins and amino acids in the pathogenesis of insulin resistance. Annu. Rev. Nutr. 27, 293–310 (2007). 17666010 10.1146/annurev.nutr.25.050304.092545

[b7] Um S. H., D'Alessio D. & Thomas G. Nutrient overload, insulin resistance, and ribosomal protein S6 kinase 1, S6K1. Cell Metab. 3, 393–402 (2006). 16753575 10.1016/j.cmet.2006.05.003

[b8] Taylor P. M. Role of amino acid transporters in amino acid sensing. Am. J. Clin. Nutr. 99, 223S–230S (2014). 24284439 10.3945/ajcn.113.070086 PMC3862456

[b9] Jewell J. L., Russell R. C. & Guan K. L. Amino acid signalling upstream of mTOR. Nat. Rev. Mol. Cell Biol. 14, 133–139 (2013). 23361334 10.1038/nrm3522 PMC3988467

[b10] Wullschleger S., Loewith R. & Hall M. N. TOR signaling in growth and metabolism. Cell 124, 471–484 (2006). 16469695 10.1016/j.cell.2006.01.016

[b11] Laplante M. & Sabatini D. M. mTOR signaling in growth control and disease. Cell 149, 274–293 (2012). 22500797 10.1016/j.cell.2012.03.017 PMC3331679

[b12] Zoncu R., Efeyan A. & Sabatini D. M. mTOR: from growth signal integration to cancer, diabetes and ageing. Nat. Rev. Mol. Cell. Biol. 12, 21–35 (2011). 21157483 10.1038/nrm3025 PMC3390257

[b13] Caballero B., Finer N. & Wurtman R. J. Plasma amino acids and insulin levels in obesity: response to carbohydrate intake and tryptophan supplements. Metabolism 37, 672–676 (1988). 3290625 10.1016/0026-0495(88)90089-3

[b14] Newgard C. B. *et al.* A branched-chain amino acid-related metabolic signature that differentiates obese and lean humans and contributes to insulin resistance. Cell Metab. 9, 311–326 (2009). 19356713 10.1016/j.cmet.2009.02.002 PMC3640280

[b15] Korsheninnikova E. *et al.* Sustained activation of the mammalian target of rapamycin nutrient sensing pathway is associated with hepatic insulin resistance, but not with steatosis, in mice. Diabetologia 49, 3049–3057 (2006). 17006666 10.1007/s00125-006-0439-5

[b16] Khamzina L., Veilleux A., Bergeron S. & Marette A. Increased activation of the mammalian target of rapamycin pathway in liver and skeletal muscle of obese rats: possible involvement in obesity-linked insulin resistance. Endocrinology 146, 1473–1481 (2005). 15604215 10.1210/en.2004-0921

[b17] Cornu M. *et al.* Hepatic mTORC1 controls locomotor activity, body temperature, and lipid metabolism through FGF21. Proc. Natl Acad. Sci. USA 111, 11592–11599 (2014). 25082895 10.1073/pnas.1412047111 PMC4136616

[b18] Kenerson H. L., Yeh M. M. & Yeung R. S. Tuberous sclerosis complex-1 deficiency attenuates diet-induced hepatic lipid accumulation. PLoS ONE 6, e18075 (2011). 21479224 10.1371/journal.pone.0018075 PMC3066210

[b19] Yecies J. L. *et al.* Akt stimulates hepatic SREBP1c and lipogenesis through parallel mTORC1-dependent and independent pathways. Cell Metab. 14, 21–32 (2011). 21723501 10.1016/j.cmet.2011.06.002 PMC3652544

[b20] Sengupta S., Peterson T. R., Laplante M., Oh S. & Sabatini D. M. mTORC1 controls fasting-induced ketogenesis and its modulation by ageing. Nature 468, 1100–1104 (2010). 21179166 10.1038/nature09584

[b21] Yamada T. *et al.* Signals from intra-abdominal fat modulate insulin and leptin sensitivity through different mechanisms: neuronal involvement in food-intake regulation. Cell Metab. 3, 223–229 (2006). 16517409 10.1016/j.cmet.2006.02.001

[b22] Seo J. *et al.* Atf4 regulates obesity, glucose homeostasis, and energy expenditure. Diabetes 58, 2565–2573 (2009). 19690063 10.2337/db09-0335 PMC2768187

[b23] Yen C. L. *et al.* Deficiency of the intestinal enzyme acyl CoA:monoacylglycerol acyltransferase-2 protects mice from metabolic disorders induced by high-fat feeding. Nat. Med. 15, 442–446 (2009). 19287392 10.1038/nm.1937 PMC2786494

[b24] Adachi H. *et al.* Angptl4 deficiency decreases serum triglyceride levels in low-density lipoprotein receptor knockout mice and streptozotocin-induced diabetic mice. Biochem. Biophys. Res. Commun. 409, 177–180 (2011). 21549101 10.1016/j.bbrc.2011.04.110

[b25] Lookene A., Skottova N. & Olivecrona G. Interactions of lipoprotein lipase with the active-site inhibitor tetrahydrolipstatin (Orlistat). Eur. J. Biochem. 222, 395–403 (1994). 8020477 10.1111/j.1432-1033.1994.tb18878.x

[b26] Kridel S. J., Axelrod F., Rozenkrantz N. & Smith J. W. Orlistat is a novel inhibitor of fatty acid synthase with antitumor activity. Cancer Res. 64, 2070–2075 (2004). 15026345 10.1158/0008-5472.can-03-3645

[b27] Preiss-Landl K., Zimmermann R., Hammerle G. & Zechner R. Lipoprotein lipase: the regulation of tissue specific expression and its role in lipid and energy metabolism. Curr. Opin. Lipidol. 13, 471–481 (2002). 12352010 10.1097/00041433-200210000-00002

[b28] Wang H. & Eckel R. H. Lipoprotein lipase: from gene to obesity. Am. J. Physiol. Endocrinol. Metab. 297, E271–E288 (2009). 19318514 10.1152/ajpendo.90920.2008

[b29] Chatterjee C. & Sparks D. L. Hepatic lipase, high density lipoproteins, and hypertriglyceridemia. Am. J. Pathol. 178, 1429–1433 (2011). 21406176 10.1016/j.ajpath.2010.12.050 PMC3078429

[b30] Huttunen J. K., Ehnholm C., Kinnunen P. K. & Nikkila E. A. An immunochemical method for the selective measurement of two triglyceride lipases in human postheparin plasma. Clin. Chim. Acta 63, 335–347 (1975). 240522 10.1016/0009-8981(75)90055-8

[b31] Sancak Y. *et al.* Ragulator-Rag complex targets mTORC1 to the lysosomal surface and is necessary for its activation by amino acids. Cell 141, 290–303 (2010). 20381137 10.1016/j.cell.2010.02.024 PMC3024592

[b32] Ono H. *et al.* Activation of hypothalamic S6 kinase mediates diet-induced hepatic insulin resistance in rats. J. Clin. Invest. 118, 2959–2968 (2008). 18618016 10.1172/JCI34277 PMC2447927

[b33] Ranganathan G., Pokrovskaya I., Ranganathan S. & Kern P. A. Role of A kinase anchor proteins in the tissue-specific regulation of lipoprotein lipase. Mol. Endocrinol. 19, 2527–2534 (2005). 15961507 10.1210/me.2005-0144

[b34] Trayhurn P., Duncan J. S. & Rayner D. V. Acute cold-induced suppression of ob (obese) gene expression in white adipose tissue of mice: mediation by the sympathetic system. Biochem. J. 311, (Pt 3): 729–733 (1995). 7487925 10.1042/bj3110729 PMC1136063

[b35] Uno K. *et al.* Hepatic peroxisome proliferator-activated receptor-γ-fat-specific protein 27 pathway contributes to obesity-related hypertension via afferent vagal signals. Eur. Heart J. 33, 1279–1289 (2012). 21825308 10.1093/eurheartj/ehr265

[b36] Porstmann T. *et al.* SREBP activity is regulated by mTORC1 and contributes to Akt-dependent cell growth. Cell Metab. 8, 224–236 (2008). 18762023 10.1016/j.cmet.2008.07.007 PMC2593919

[b37] Lu B. *et al.* Metabolic crosstalk: molecular links between glycogen and lipid metabolism in obesity. Diabetes 63, 2935–2948 (2014). 24722244 10.2337/db13-1531 PMC4141363

[b38] Li S., Brown M. S. & Goldstein J. L. Bifurcation of insulin signaling pathway in rat liver: mTORC1 required for stimulation of lipogenesis, but not inhibition of gluconeogenesis. Proc. Natl Acad. Sci. USA 107, 3441–3446 (2010). 20133650 10.1073/pnas.0914798107 PMC2840492

[b39] Singh R. *et al.* Autophagy regulates lipid metabolism. Nature 458, 1131–1135 (2009). 19339967 10.1038/nature07976 PMC2676208

[b40] Duvel K. *et al.* Activation of a metabolic gene regulatory network downstream of mTOR complex 1. Mol. Cell 39, 171–183 (2010). 20670887 10.1016/j.molcel.2010.06.022 PMC2946786

[b41] Peterson T. R. *et al.* mTOR complex 1 regulates lipin 1 localization to control the SREBP pathway. Cell 146, 408–420 (2011). 21816276 10.1016/j.cell.2011.06.034 PMC3336367

[b42] Li S. *et al.* Role of S6K1 in regulation of SREBP1c expression in the liver. Biochem. Biophys. Res. Commun. 412, 197–202 (2011). 21806970 10.1016/j.bbrc.2011.07.038

[b43] Wan M. *et al.* Postprandial hepatic lipid metabolism requires signaling through Akt2 independent of the transcription factors FoxA2, FoxO1, and SREBP1c. Cell Metab. 14, 516–527 (2011). 21982711 10.1016/j.cmet.2011.09.001 PMC3190164

[b44] Bakan I. & Laplante M. Connecting mTORC1 signaling to SREBP-1 activation. Curr. Opin. Lipidol. 23, 226–234 (2012). 22449814 10.1097/MOL.0b013e328352dd03

[b45] Torii K. & Niijima A. Effect of lysine on afferent activity of the hepatic branch of the vagus nerve in normal and L-lysine-deficient rats. Physiol. Behav. 72, 685–690 (2001). 11337000 10.1016/s0031-9384(01)00426-7

[b46] Tome D., Schwarz J., Darcel N. & Fromentin G. Protein, amino acids, vagus nerve signaling, and the brain. Am. J. Clin. Nutr. 90, 838S–843S (2009). 19640948 10.3945/ajcn.2009.27462W

[b47] Brunzell J. D. Clinical practice. Hypertriglyceridemia. N. Engl. J. Med. 357, 1009–1017 (2007). 17804845 10.1056/NEJMcp070061

[b48] Mazzone T., Chait A. & Plutzky J. Cardiovascular disease risk in type 2 diabetes mellitus: insights from mechanistic studies. Lancet 371, 1800–1809 (2008). 18502305 10.1016/S0140-6736(08)60768-0 PMC2774464

[b49] Levak-Frank S. *et al.* Muscle-specific overexpression of lipoprotein lipase causes a severe myopathy characterized by proliferation of mitochondria and peroxisomes in transgenic mice. J. Clin. Invest. 96, 976–986 (1995). 7635990 10.1172/JCI118145 PMC185285

[b50] Lee J. H. *et al.* The transcription factor cyclic AMP-responsive element-binding protein H regulates triglyceride metabolism. Nat. Med. 17, 812–815 (2011). 21666694 10.1038/nm.2347 PMC3374483

[b51] Kovar J. & Adamkova V. Lipoprotein lipase activity determined *in vivo* is lower in carriers of apolipoprotein A-V gene variants 19W and -1131C. Physiol. Res. 57, 555–561 (2008). 17705673 10.33549/physiolres.931245

[b52] Henderson H., Leisegang F., Hassan F., Hayden M. & Marais D. A novel Glu421Lys substitution in the lipoprotein lipase gene in pregnancy-induced hypertriglyceridemic pancreatitis. Clin. Chim. Acta 269, 1–12 (1998). 9498099 10.1016/s0009-8981(97)00144-7

[b53] Augustus A. *et al.* Cardiac-specific knock-out of lipoprotein lipase alters plasma lipoprotein triglyceride metabolism and cardiac gene expression. J. Biol. Chem. 279, 25050–25057 (2004). 15028738 10.1074/jbc.M401028200

[b54] Antras J., Lasnier F. & Pairault J. Beta-adrenergic-cyclic AMP signalling pathway modulates cell function at the transcriptional level in 3T3-F442A adipocytes. Mol. Cell. Endocrinol. 82, 183–190 (1991). 1665451 10.1016/0303-7207(91)90030-v

[b55] Raynolds M. V. *et al.* Lipoprotein lipase gene expression in rat adipocytes is regulated by isoproterenol and insulin through different mechanisms. Mol. Endocrinol. 4, 1416–1422 (1990). 2233752 10.1210/mend-4-9-1416

[b56] Inoki K., Kim J. & Guan K. L. AMPK and mTOR in cellular energy homeostasis and drug targets. Annu. Rev. Pharmacol. Toxicol. 52, 381–400 (2012). 22017684 10.1146/annurev-pharmtox-010611-134537

[b57] Rabinowitz J. D. & White E. Autophagy and metabolism. Science 330, 1344–1348 (2010). 21127245 10.1126/science.1193497 PMC3010857

[b58] Schwarz J., Tome D., Baars A., Hooiveld G. J. & Muller M. Dietary protein affects gene expression and prevents lipid accumulation in the liver in mice. PLoS ONE 7, e47303 (2012). 23110065 10.1371/journal.pone.0047303 PMC3479095

[b59] Freudenberg A., Petzke K. J. & Klaus S. Comparison of high-protein diets and leucine supplementation in the prevention of metabolic syndrome and related disorders in mice. J. Nutr. Biochem. 23, 1524–1530 (2012). 22405695 10.1016/j.jnutbio.2011.10.005

[b60] Bortolotti M., Dubuis J., Schneiter P. & Tappy L. Effects of dietary protein on lipid metabolism in high fructose fed humans. Clin. Nutr. 31, 238–245 (2012). 22019201 10.1016/j.clnu.2011.09.011

[b61] Kahn S. E., Hull R. L. & Utzschneider K. M. Mechanisms linking obesity to insulin resistance and type 2 diabetes. Nature 444, 840–846 (2006). 17167471 10.1038/nature05482

[b62] Hyde R., Cwiklinski E. L., MacAulay K., Taylor P. M. & Hundal H. S. Distinct sensor pathways in the hierarchical control of SNAT2, a putative amino acid transceptor, by amino acid availability. J. Biol. Chem. 282, 19788–19798 (2007). 17488712 10.1074/jbc.M611520200

[b63] Gonzalez-Gonzalez I. M., Cubelos B., Gimenez C. & Zafra F. Immunohistochemical localization of the amino acid transporter SNAT2 in the rat brain. Neuroscience 130, 61–73 (2005). 15561425 10.1016/j.neuroscience.2004.09.023

[b64] Hatanaka T., Hatanaka Y. & Setou M. Regulation of amino acid transporter ATA2 by ubiquitin ligase Nedd4-2. J. Biol. Chem. 281, 35922–35930 (2006). 17003038 10.1074/jbc.M606577200

[b65] Usui S., Hara Y., Hosaki S. & Okazaki M. A new on-line dual enzymatic method for simultaneous quantification of cholesterol and triglycerides in lipoproteins by HPLC. J. Lipid Res. 43, 805–814 (2002). 11971952

[b66] Qu S. *et al.* Effects of apoA-V on HDL and VLDL metabolism in APOC3 transgenic mice. J. Lipid Res. 48, 1476–1487 (2007). 17438339 10.1194/jlr.M600498-JLR200 PMC2665252

[b67] Altomonte J. *et al.* Foxo1 mediates insulin action on apoC-III and triglyceride metabolism. J. Clin. Invest. 114, 1493–1503 (2004). 15546000 10.1172/JCI19992 PMC525736

[b68] Trost Z., Sok M., Marc J. & Cerne D. Increased lipoprotein lipase activity in non-small cell lung cancer tissue predicts shorter patient survival. Arch. Med. Res. 40, 364–368 (2009). 19766899 10.1016/j.arcmed.2009.05.001

[b69] Yen F. T. *et al.* Lipolysis stimulated lipoprotein receptor: a novel molecular link between hyperlipidemia, weight gain, and atherosclerosis in mice. J. Biol. Chem. 283, 25650–25659 (2008). 18644789 10.1074/jbc.M801027200

[b70] Shigeta Y., Nakamura K., Hoshi M., Kim M. & Abe H. Effect of dextran sulfate on plasma lipoprotein lipase activity in obese subjects and diabetic patients. Diabetes 16, 238–241 (1967). 6023166 10.2337/diab.16.4.238

