# Supplementary material for: A hepatic amino acid/mTOR/S6K-dependent signalling pathway modulates systemic lipid metabolism via neuronal signals
Source: Nat Commun. 2015 Aug 13;6:7940. doi: 10.1038/ncomms8940 (PMC4557134; doi:10.1038/ncomms8940)
Supplement: Supplementary Information — Supplementary Figures 1-19 and Supplementary Tables 1-2 [file ncomms8940-s1.pdf]

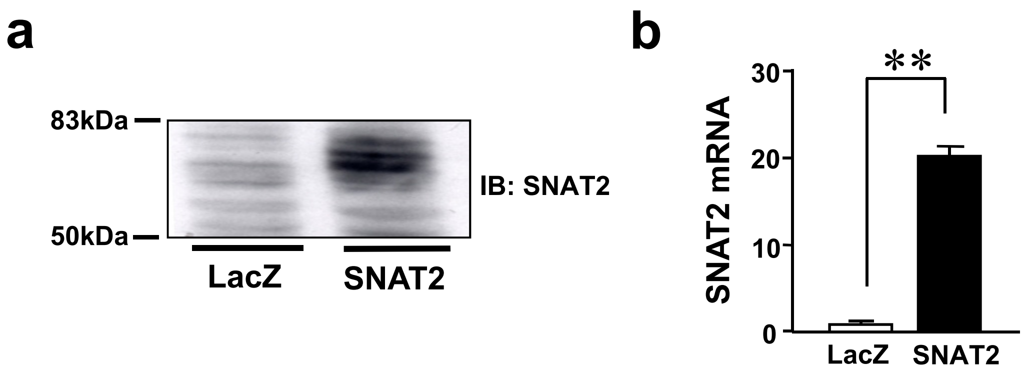

Supplementary Figure 1. Analyses of adenoviral SNAT2 expression in the livers of SNAT2-mice

SNAT2 (black bar) or LacZ (white bar) adenovirus was administered to high fat diet-fed C57BL/6 mice. (a) Liver extracts were immunoblotted with anti-SNAT2 antibody on day 5 after adenoviral administration. The representative images derived from at least duplicate experiments were displayed. (b) Hepatic SNAT2 mRNA expressions were examined on day 5 after adenoviral administration (*n*=5-7). Data are presented as means±SD. ***P* < 0.01 by the unpaired *t* test.

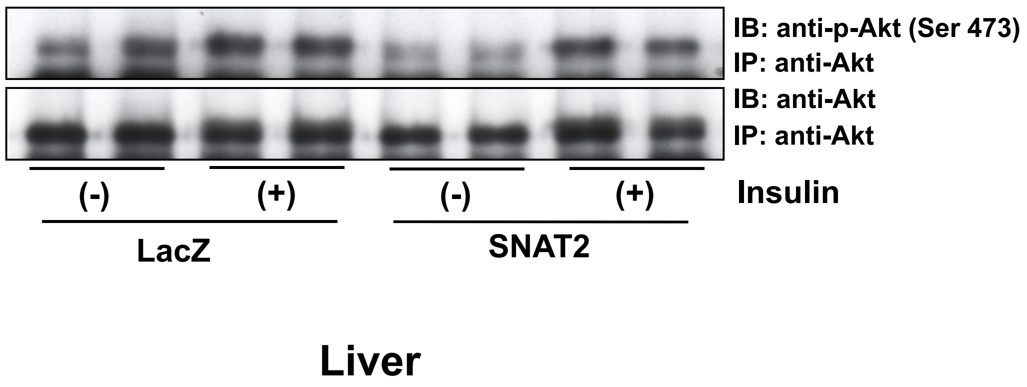

Supplementary Figure 2. Insulin-stimulated serine phosphorylation of Akt in the livers of SNAT2-mice

Mice fasted 16 hours were injected intravenously with insulin or vehicle alone. Their livers were removed 300 s later and lysates were immunoprecipitated with anti-Akt antibody, followed by SDS-PAGE and immunoblotting with anti-phospho-serine 473 of Akt antibody and anti-Akt antibody.

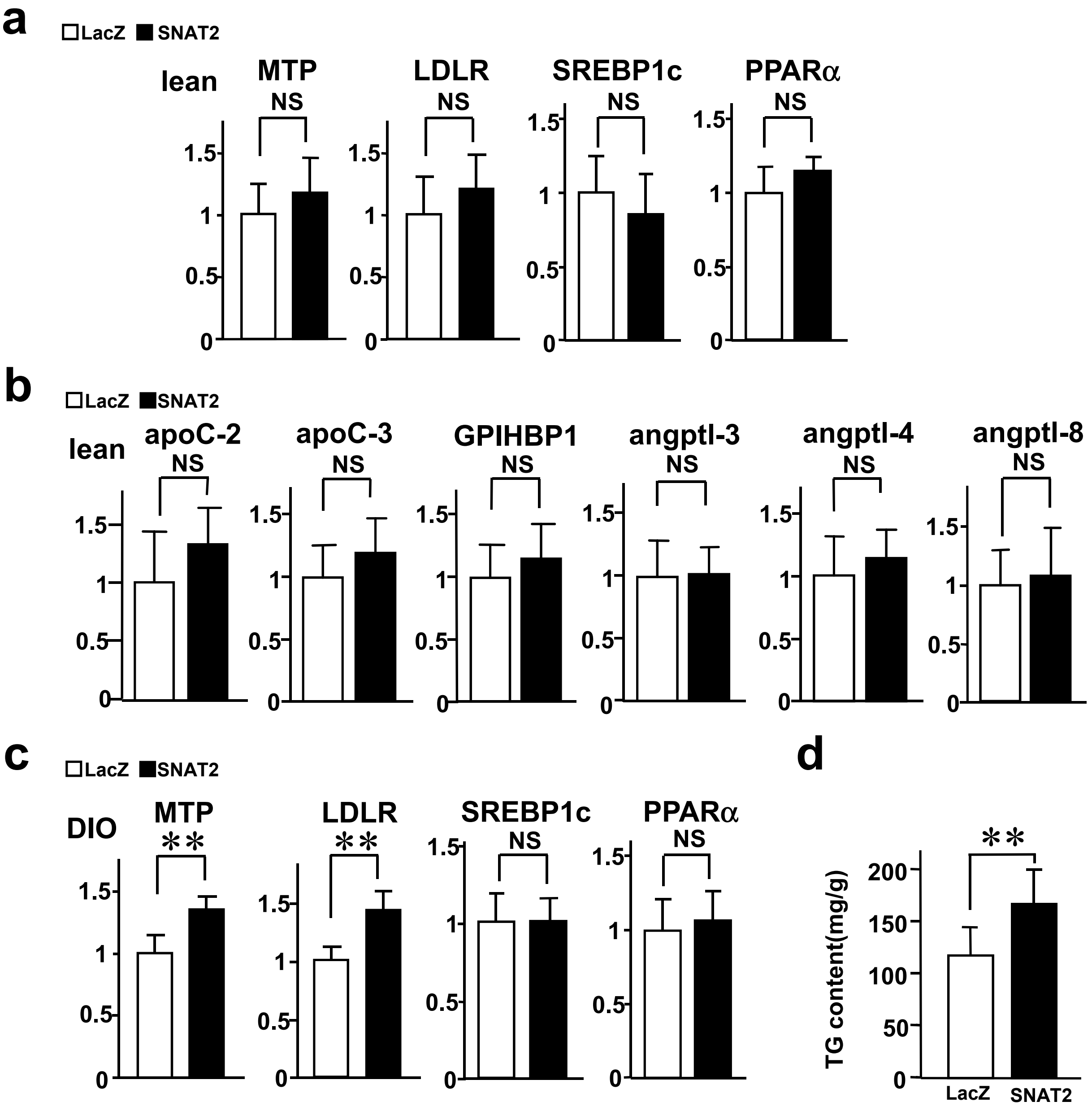

Supplementary Figure 3. Analysis of hepatic expressions of molecules related to hepatic lipid metabolism and serum TG-hydrolysis in SNAT2-mice

SNAT2 (black bars) or LacZ (white bars) adenovirus was administered to standard chow-fed C57BL/6 mice (a-b) or high fat diet-fed C57BL/6 mice (DIO) (c-d). (a, b and c) mRNA expression levels of several genes in the liver were examined on day 5 after adenoviral administration (a; n=5-7, b; n=6-7, c; n=5-7). (d) Hepatic TG contents were examined on day 5 after adenovirus administration (n=5-6). Data are presented as means±SD. **P < 0.01 by the unpaired t test.

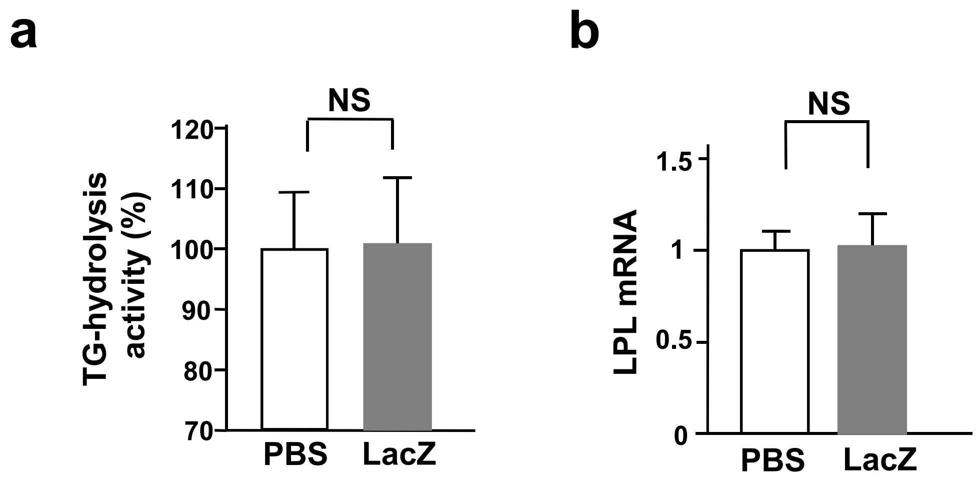

Supplementary Figure 4. Adenoviral administration itself affects neither plasma TG-hydrolysis activity nor LPL mRNA expression in WAT

(a-b) LacZ (gray bar) adenovirus or PBS (white bar) was administered to standard chow-fed C57BL/6 mice. (a) Plasma TG-hydrolysis activity was measured after injection of heparin into the tail veins of mice on day 5 after adenovirus administration (n=5-7). (b) LPL mRNA expression in WAT was examined on day 5 after adenoviral administration (n=6-7). Data are presented as means±SD. Analysis by the unpaired t test.

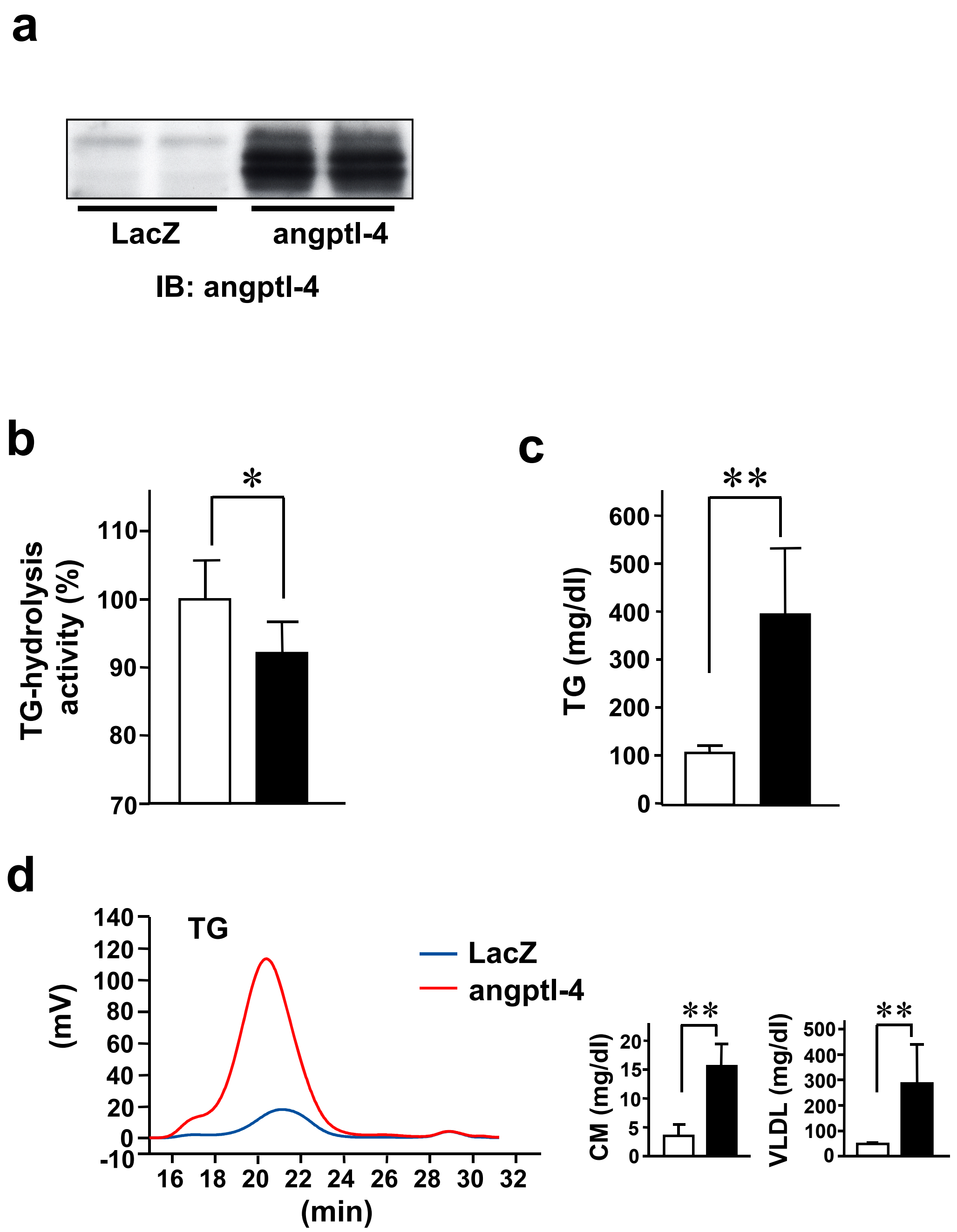

Supplementary Figure 5. Hepatic angptl-4 expression raises serum triglyceride levels by suppressing TG-hydrolysis activities

angptl-4 (black bars) or LacZ (white bars) adenovirus was administered to standard chow-fed C57BL/6 mice. (a) Liver extracts were immunoblotted with anti-angptl4 antibody on day 5 after adenoviral administration. The representative images derived from at least duplicate experiments were displayed. Plasma TG-hydrolysis activities (b) and serum TG levels in the fed state (c) were measured on day 5 after adenovirus administration (b; *n*=5, c; *n*=5-7). HPLC analyses of sera from these mice in fed states were performed (d) on day 5 after adenovirus administration. For HPLC analysis, the graphs show data from one mouse, representing each of the two groups studied. Similar results were obtained with other mice (n=5) from each experimental group. Data are presented as means±SD. *P < 0.05, **P < 0.01 by the unpaired t test.

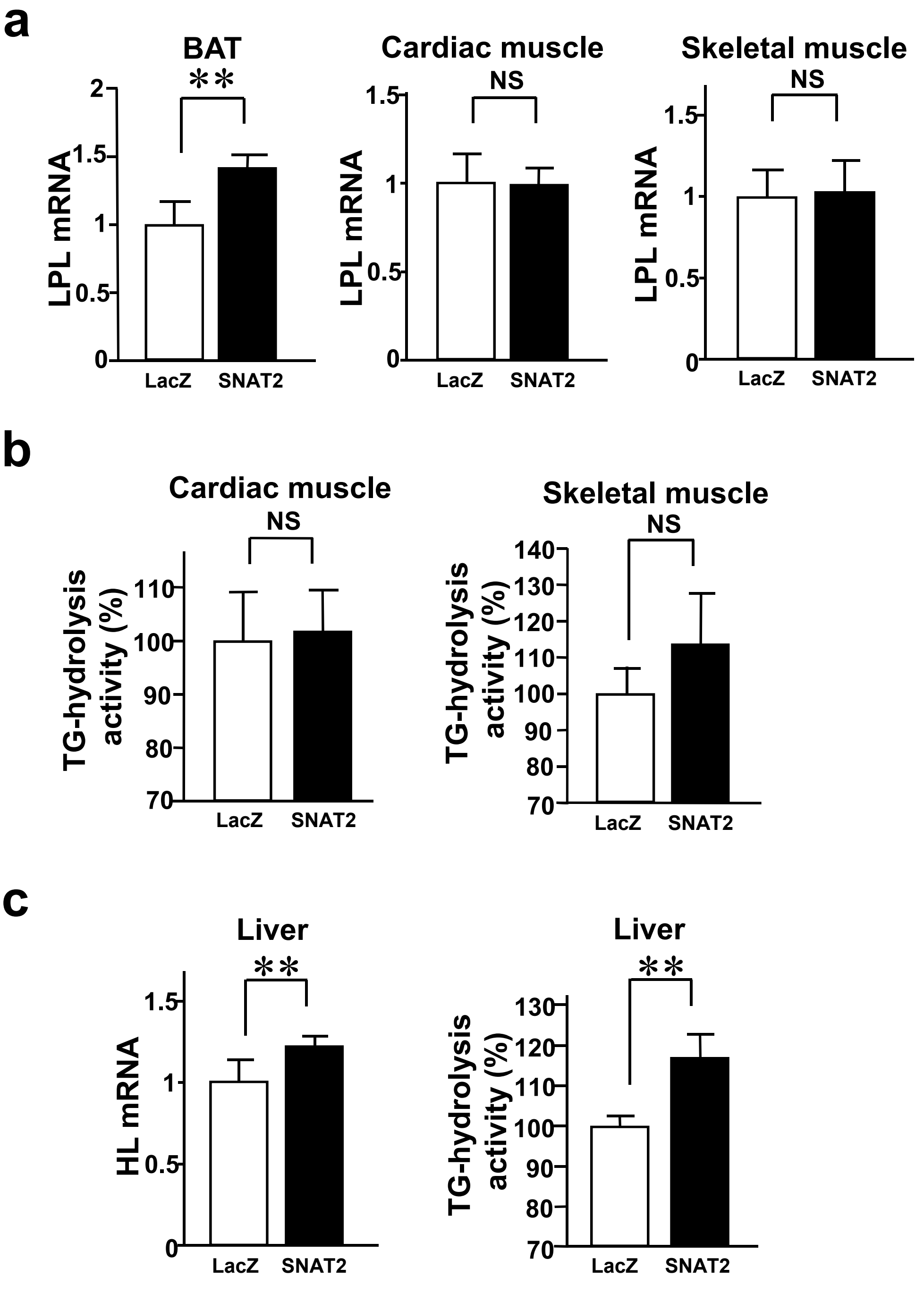

Supplementary Figure 6. Analysis of LPL expressions and TG-hydrolysis activities in several tissues, and analysis of HL expression and TG-hydrolysis activity in the livers of SNAT2-mice

SNAT2 (black bars) or LacZ (white bars) adenovirus was administered to high fat diet-fed C57BL/6 mice. (a) LPL mRNA expressions in BAT, cardiac muscle and skeletal muscle were examined on day 5 after adenoviral administration (BAT; *n*=5-7, cardiac and skeletal muscles; *n*=5-6). (b) TG-hydrolysis activities in cardiac muscle and skeletal muscle were examined on day 5 after adenoviral administration (*n*=5-6). (c) HL mRNA expression, and TG-hydrolysis activity in the liver were examined on day 5 after adenoviral administration (*n*=5-7). Data are presented as means±SD. $^{**}P < 0.01$ by the unpaired *t* test.

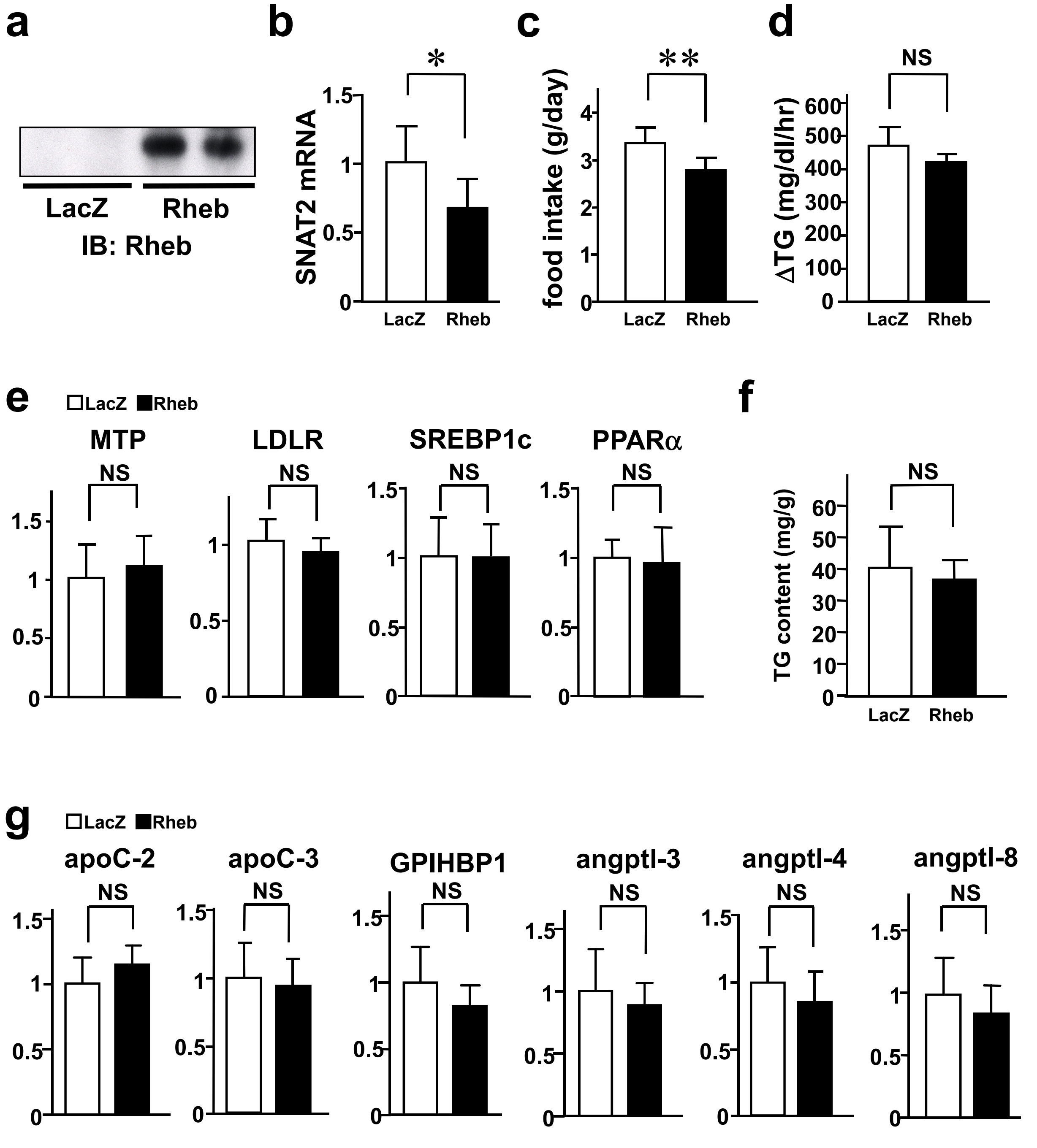

Supplementary Figure 7. Analysis of adenoviral Rheb expression in the liver, and analysis of hepatic expressions of molecules related to hepatic lipid metabolism and serum TG-hydrolysis in Rheb-mice

(a-g) Rheb (black bars) or LacZ (white bars) adenovirus was administered to standard chow-fed C57BL/6 mice. (a) Liver extracts were immunoblotted with anti-Rheb antibody on day 5 after adenoviral administration. The representative images derived from at least duplicate experiments were displayed. (b) SNAT2 mRNA expression in the liver was determined on day 5 after adenovirus administration (*n*=5-6). (c) Food intakes were measured for 5 days after adenovirus administration (*n*=5). (d) The hepatic TG secretion rates were determined by measuring serum TG levels after injection of Triton WR-1339 into the tail veins of 6 hr-fasted mice 5 days after adenovirus administration (*n*=4-5). (e and g) mRNA expression levels of several genes in the liver were examined on day 5 after adenoviral administration (e; *n*=5-7, g; *n*=5-11). (f) Hepatic TG contents were examined on day 5 after adenovirus administration (*n*=5-6). Data are presented as means±SD. **P* < 0.05, ***P* < 0.01 by the unpaired *t* test.

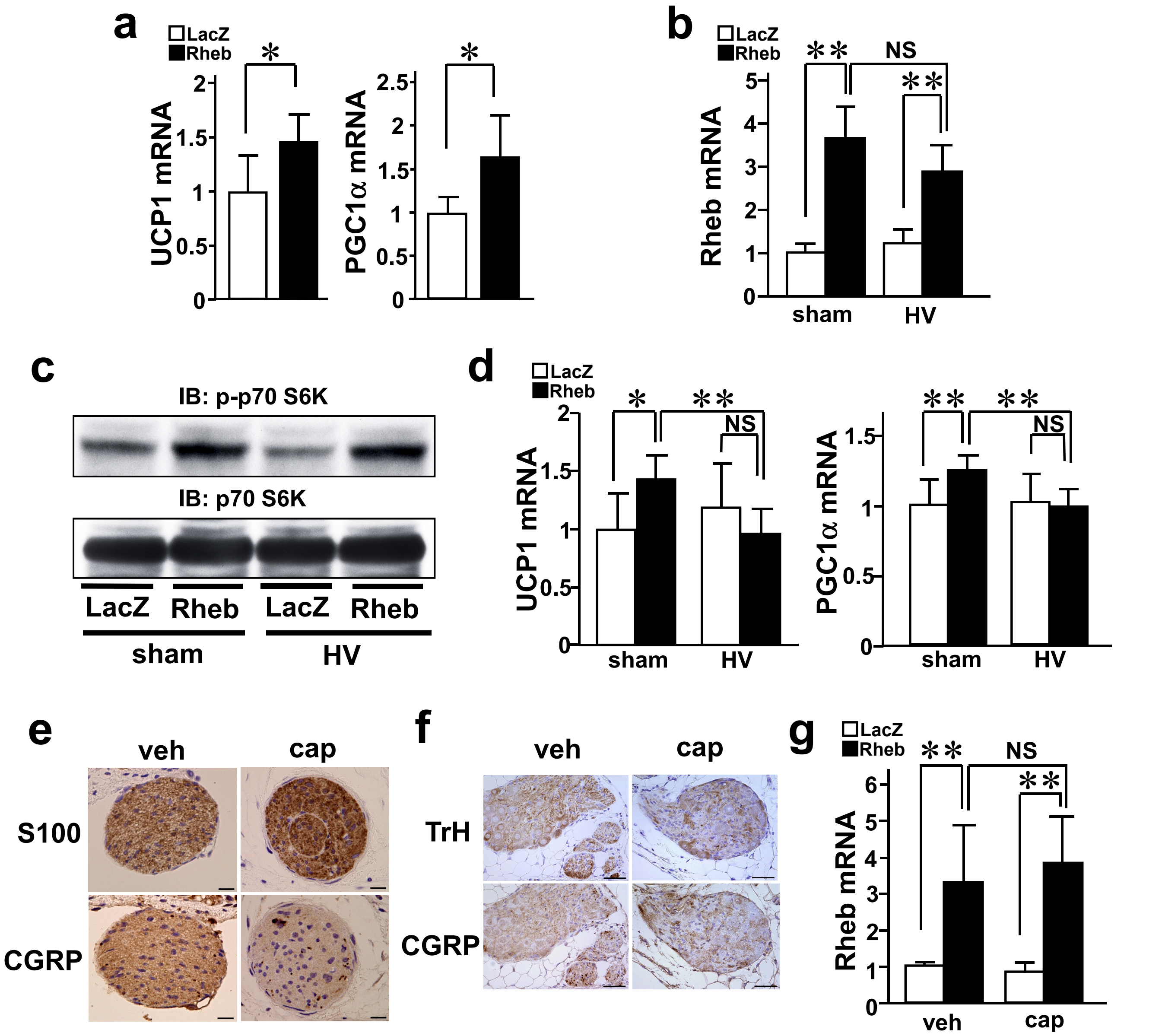

Supplementary Figure 8. HV blocks the increases of specific gene expressions in WAT up-regulated by hepatic Rheb expression, and capsaicin treatment selectively blocks the afferent hepatic vagus

(a) Rheb (black bars) or LacZ (white bars) adenovirus was administered to standard chow-fed C57BL/6 mice. UCP1 and PGC1α mRNA expressions in WAT were examined on day 5 after adenovirus administration (*n*=5-6). (b-d) Standard chow-fed C57BL/6 mice were subjected to sham operation (sham) or HV 7 days prior to adenoviral administration. Rheb (black bars) or LacZ (white bars) adenovirus was administered to these mice. (b) Rheb mRNA expression was examined (*n*=6-7). (c) Liver extracts were immunoblotted with anti-phospho p70-S6K or p70-S6K antibody on day 5 after adenovirus administration. The representative images derived from at least duplicate experiments were displayed. (d) UCP1 and PGC1α mRNA expressions in WAT were examined on day 5 after adenovirus administration (*n*=5-7). (e and g) Vehicle (veh) or capsaicin (cap) was applied to the hepatic vagus of standard chow-fed C57BL/6 mice 7 days prior to adenoviral administration. Rheb (black bars) or LacZ (white bars) adenovirus was then administered to these mice. (e) Immunohistochemistry of the hepatic vagus with anti-S100 (upper panels) and anti-calcitonin gene related peptide (CGRP) (lower panels) antibodies. Magnification: x400. Scale bars: 50 μm. (f) Immunohistochemistry of the splanchnic nerves with anti-tyrosine hydroxylase (TrH) (upper panels) and anti-CGRP (lower panels) antibodies after veh or cap application to the hepatic vagus of standard chow-fed C57BL/6 mice. Magnification: x400. Scale bars: 50 μm. (g) Rheb mRNA expression was examined on day 5 after adenovirus administration (*n*=6-8). Data are presented as means±SD. **P* < 0.05, ***P* < 0.01 by the unpaired *t* test.

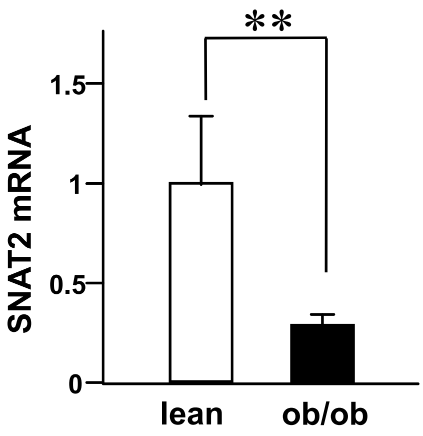

Supplementary Figure 9. Analyses of adenoviral SNAT2 expressions in the livers of lean and ob/ob mice

Measurement of hepatic expressions of SNAT2 mRNAs by quantitative RT-PCR, in standard chow-fed mice (white bar) and genetically obese ob/ob mice (black bar) (n=7-12). Data are presented as means±SD. **P < 0.01 by the unpaired t test.

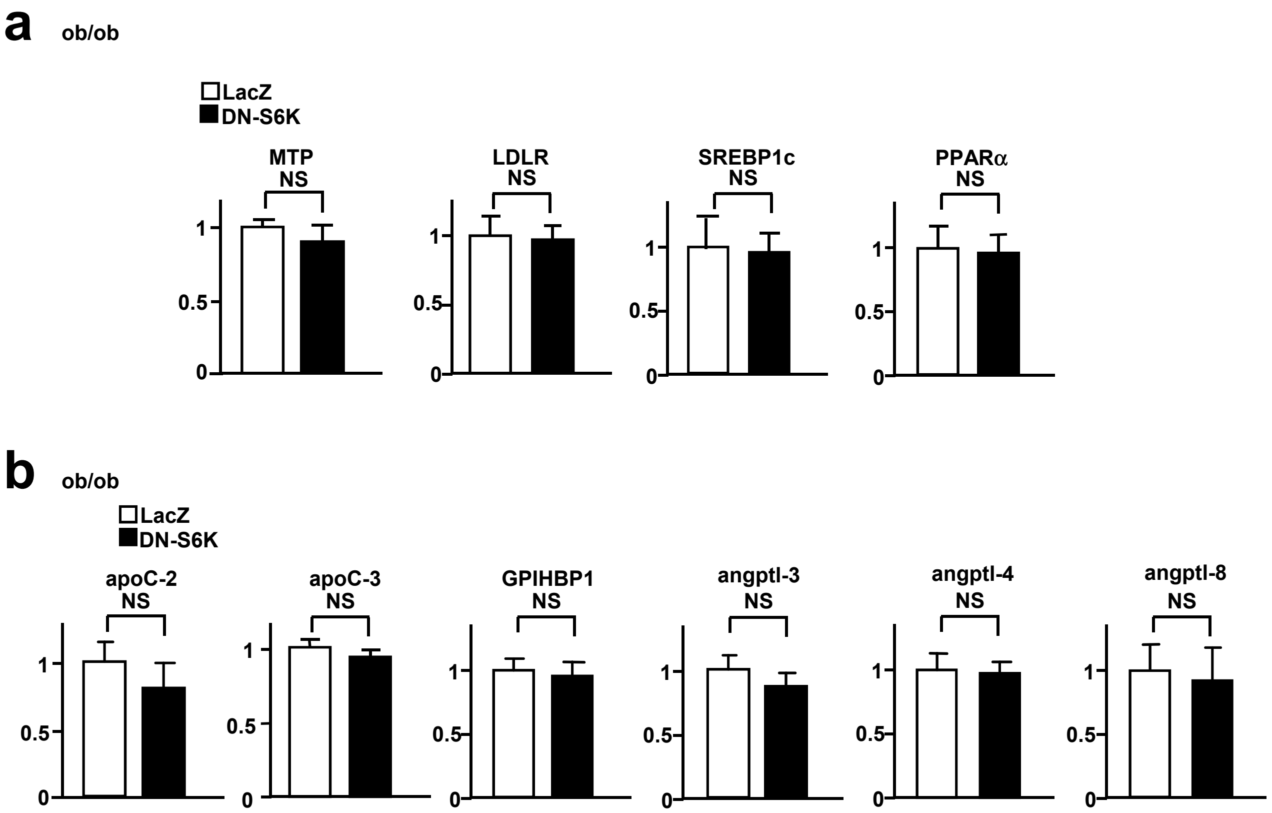

Supplementary Figure 10. Analysis of hepatic expressions of molecules related to hepatic lipid metabolism and serum TG-hydrolysis in DN-S6K-mice

DN-S6K (black bars) or LacZ (white bars) adenovirus was administered to standard chow-fed ob/ob mice. mRNA expression levels of several genes in the liver were examined on day 5 after adenoviral administration. (a-b) mRNA expression levels of several genes in the liver were examined on day 5 after adenoviral administration (a; *n*=4-5, b; *n*=4-5). Data are presented as means±SD. Analysis by the unpaired *t* test.

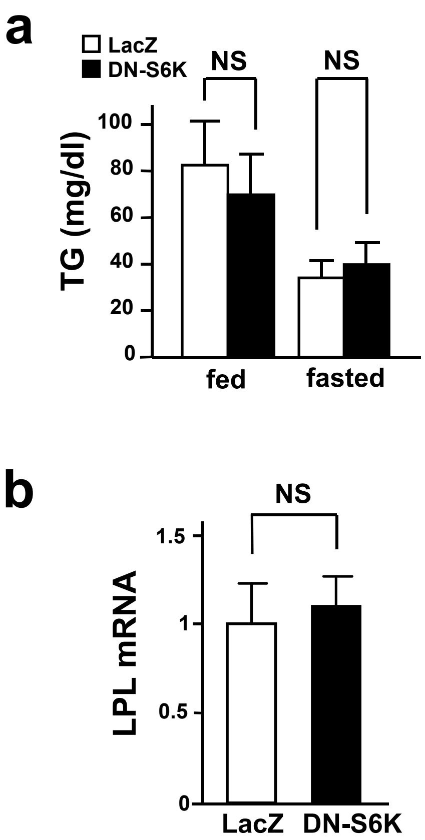

Supplementary Figure 11. Hepatic DN-S6K expression does changes neither serum TG levels nor adipose LPL expressions in lean C57BL/6 mice

(a and b) DN-S6K (black bars) or LacZ (white bars) adenovirus was administered to standard chow-fed C57BL/6 mice. Serum TG levels (a) and LPL mRNA expressions in WAT (b) were examined on day 5 after adenoviral administration (a; *n*=4-7, b; *n*=5-6). Data are presented as means±SD. Analysis by the unpaired *t* test.

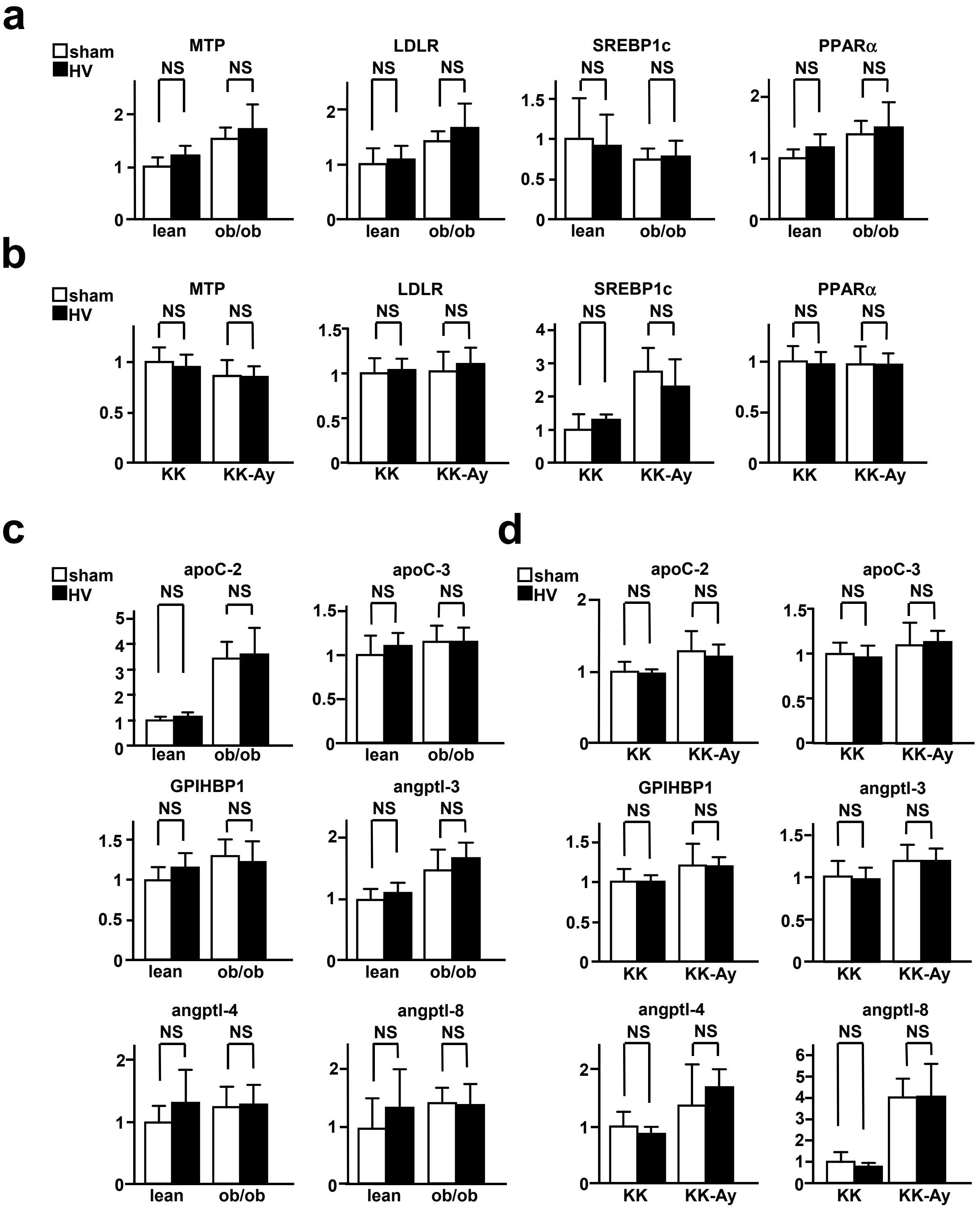

Supplementary Figure 12. Analysis of hepatic expressions of molecules related to hepatic lipid metabolism and serum TG-hydrolysis in sham- and HV-mice

(a and c) Standard chow-fed C57BL/6 and ob/ob mice were subjected to sham operation (sham) or HV. mRNA expression levels of several genes in the liver were examined on day 14 after these operations (a; *n*=6-7, c; *n*=5-7). (b and d) Standard chow-fed KK and KK-Ay mice were subjected to sham operation or HV. mRNA expression levels of several genes in the liver were examined on day 14 after these operations (b; *n*=5-8, d; *n*=6-8). Data are presented as means±SD. Analysis by the unpaired *t* test.

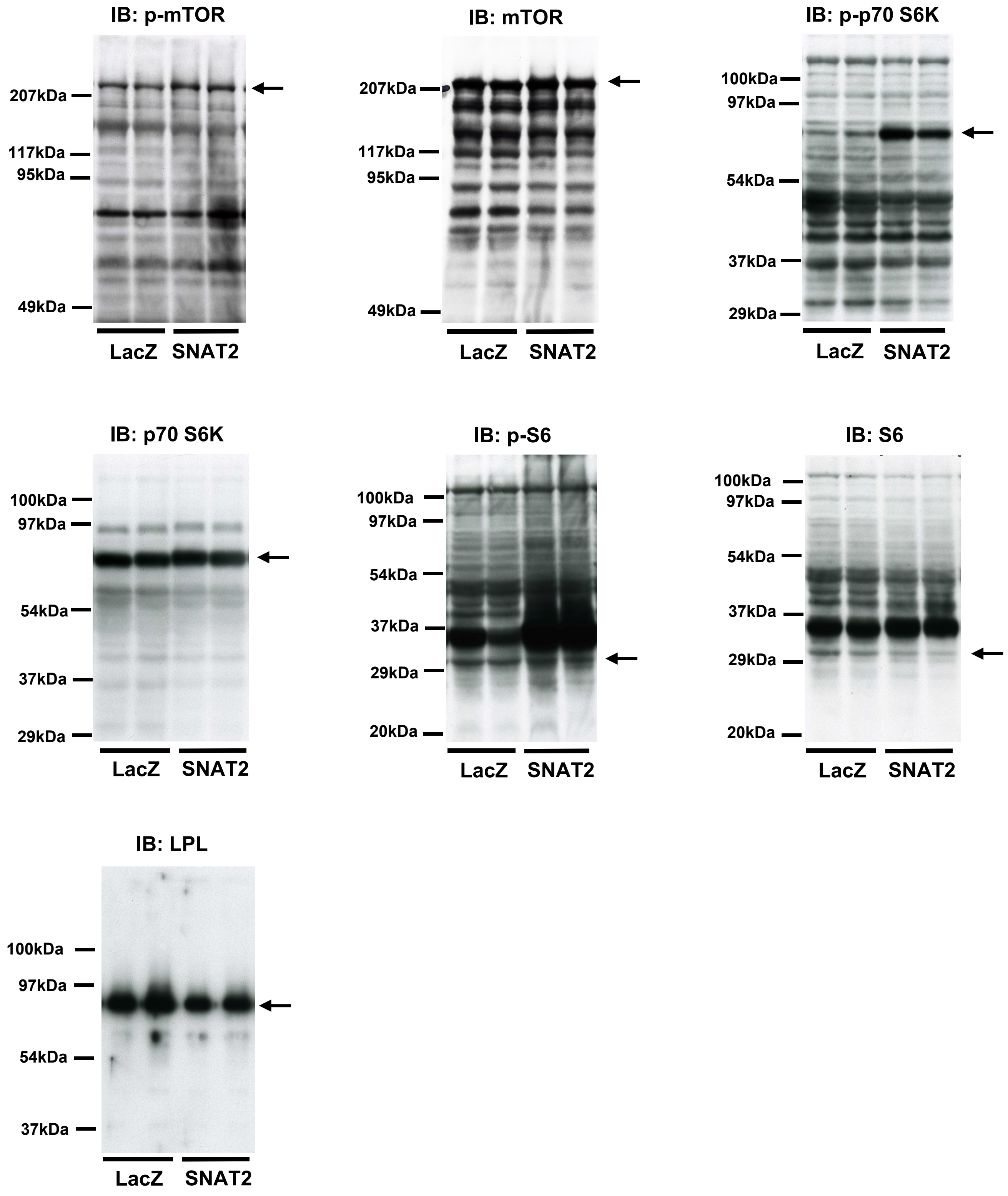

Supplementary Figure 13. Full western blots in Figure 1 and Figure 2

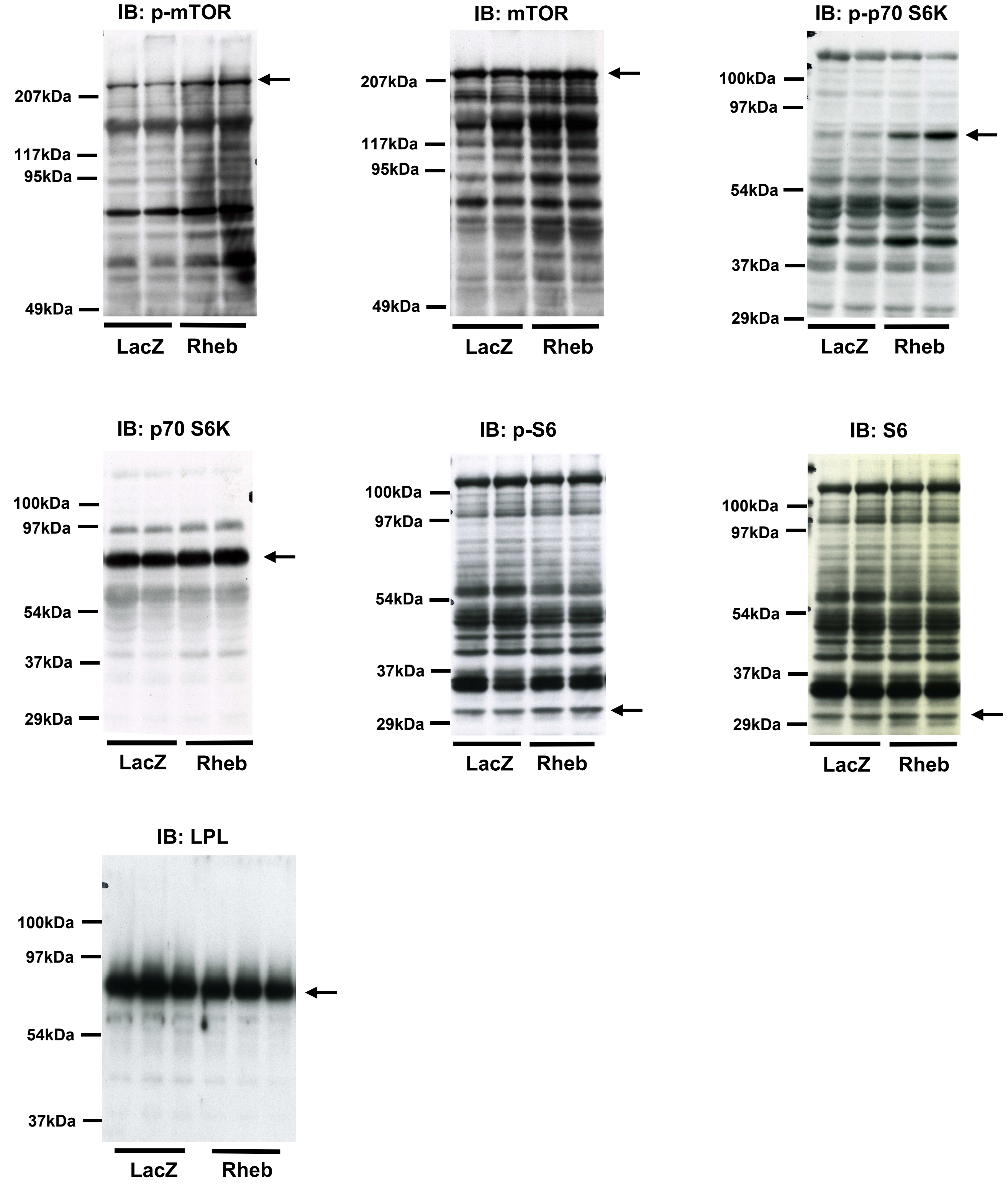

Supplementary Figure 14. Full western blots in Figure 3

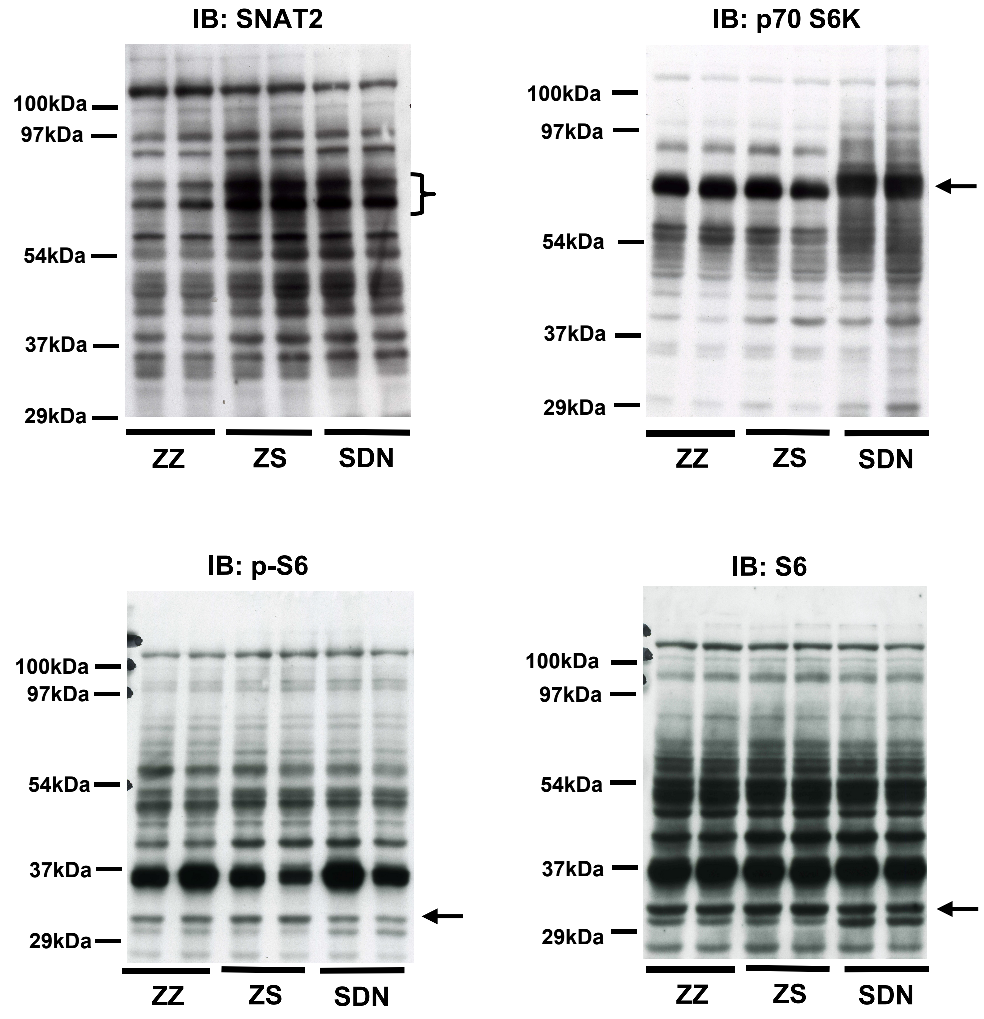

Supplementary Figure 15. Full western blots in Figure 5

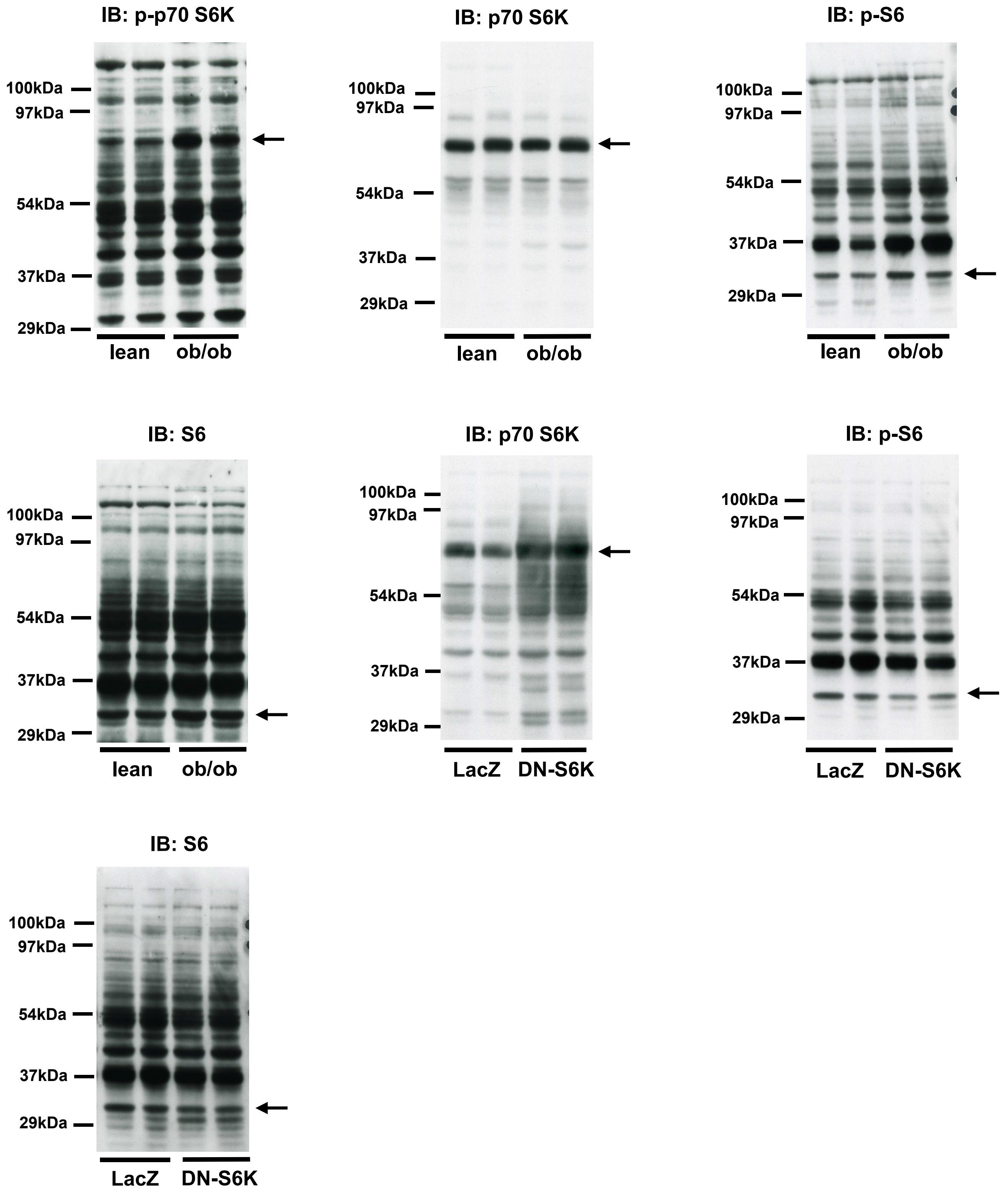

Supplementary Figure 16. Full western blots in Figure 7

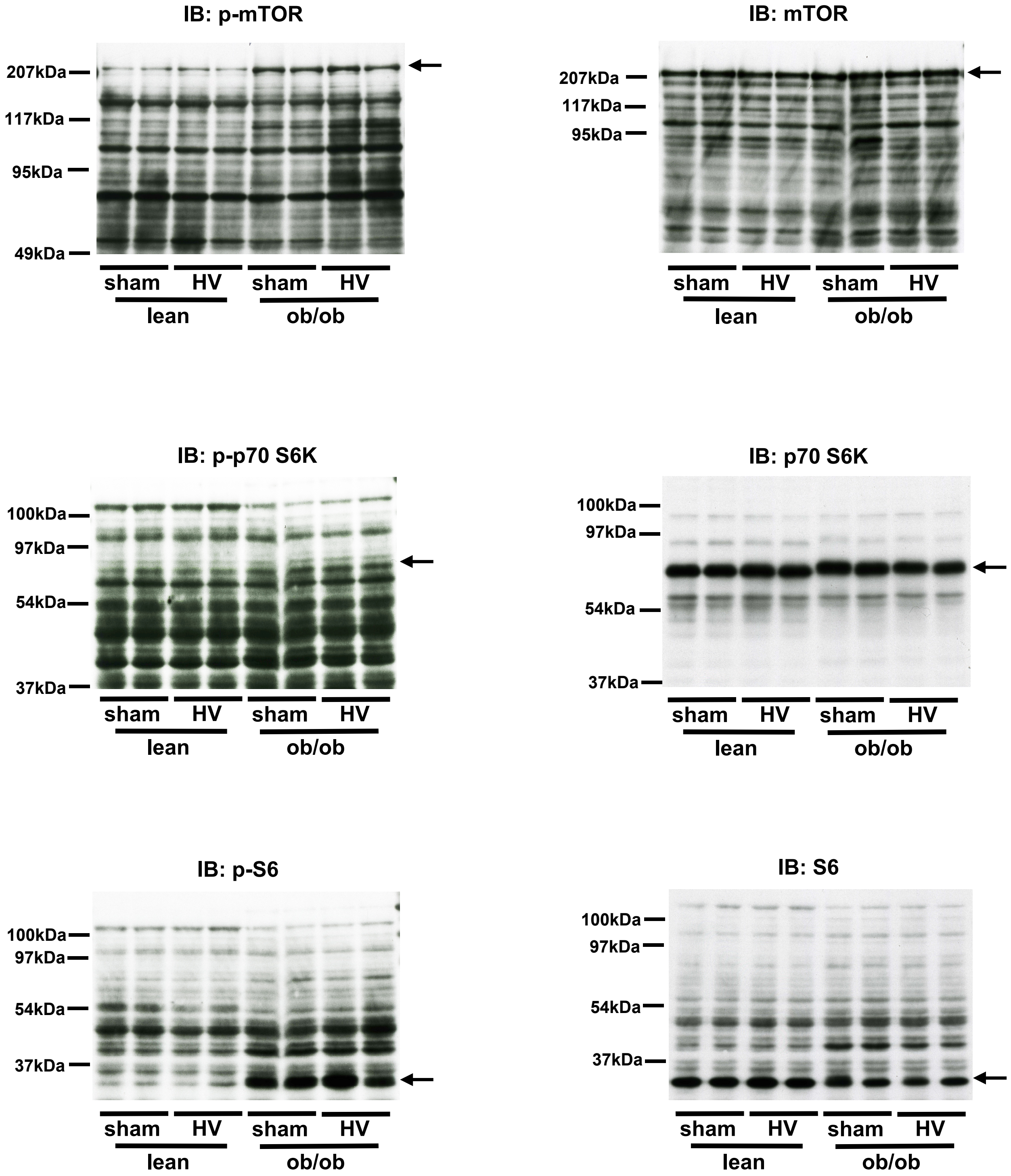

Supplementary Figure 17. Full western blots in Figure 9a

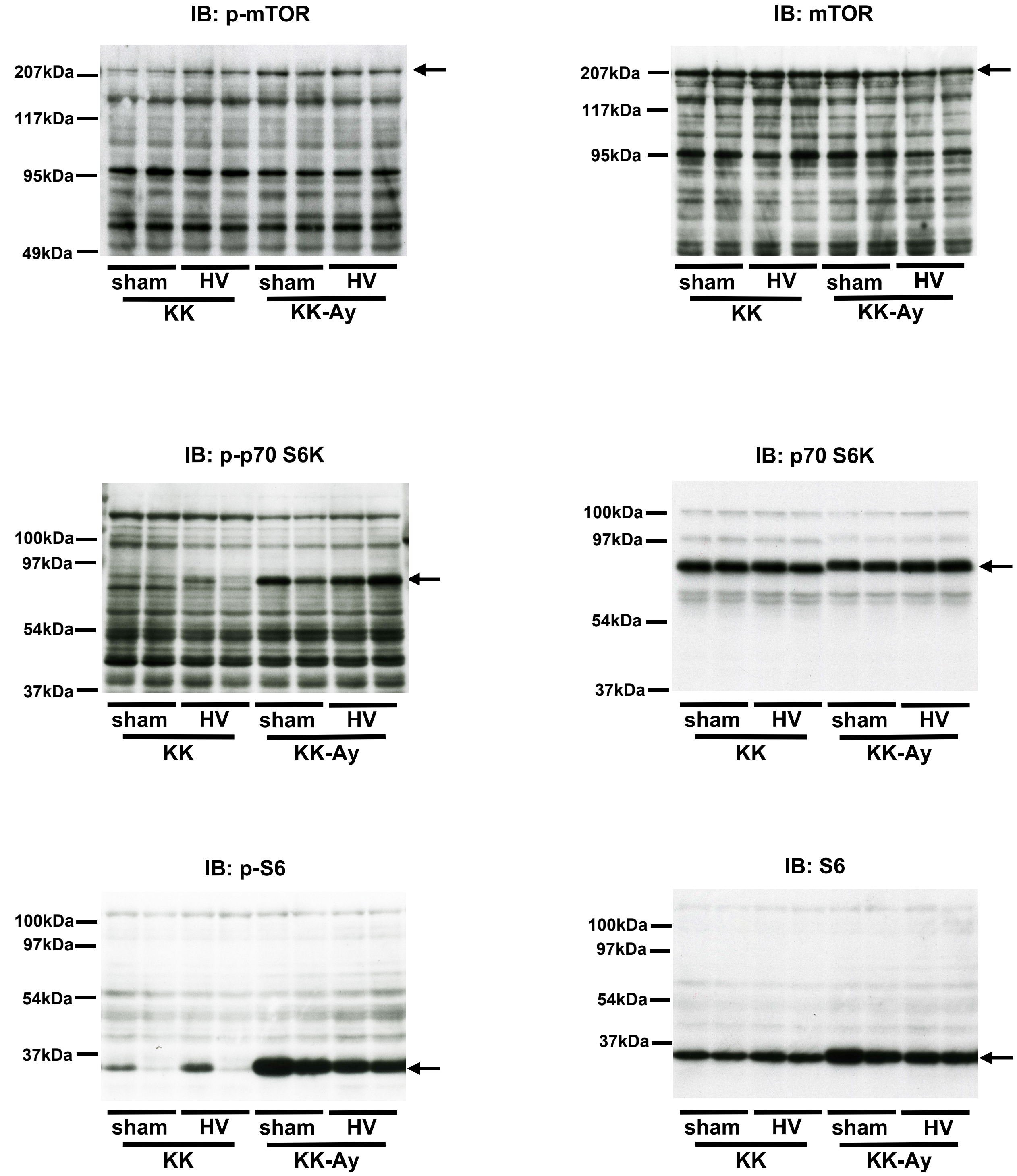

Supplementary Figure 18. Full western blots in Figure 9b

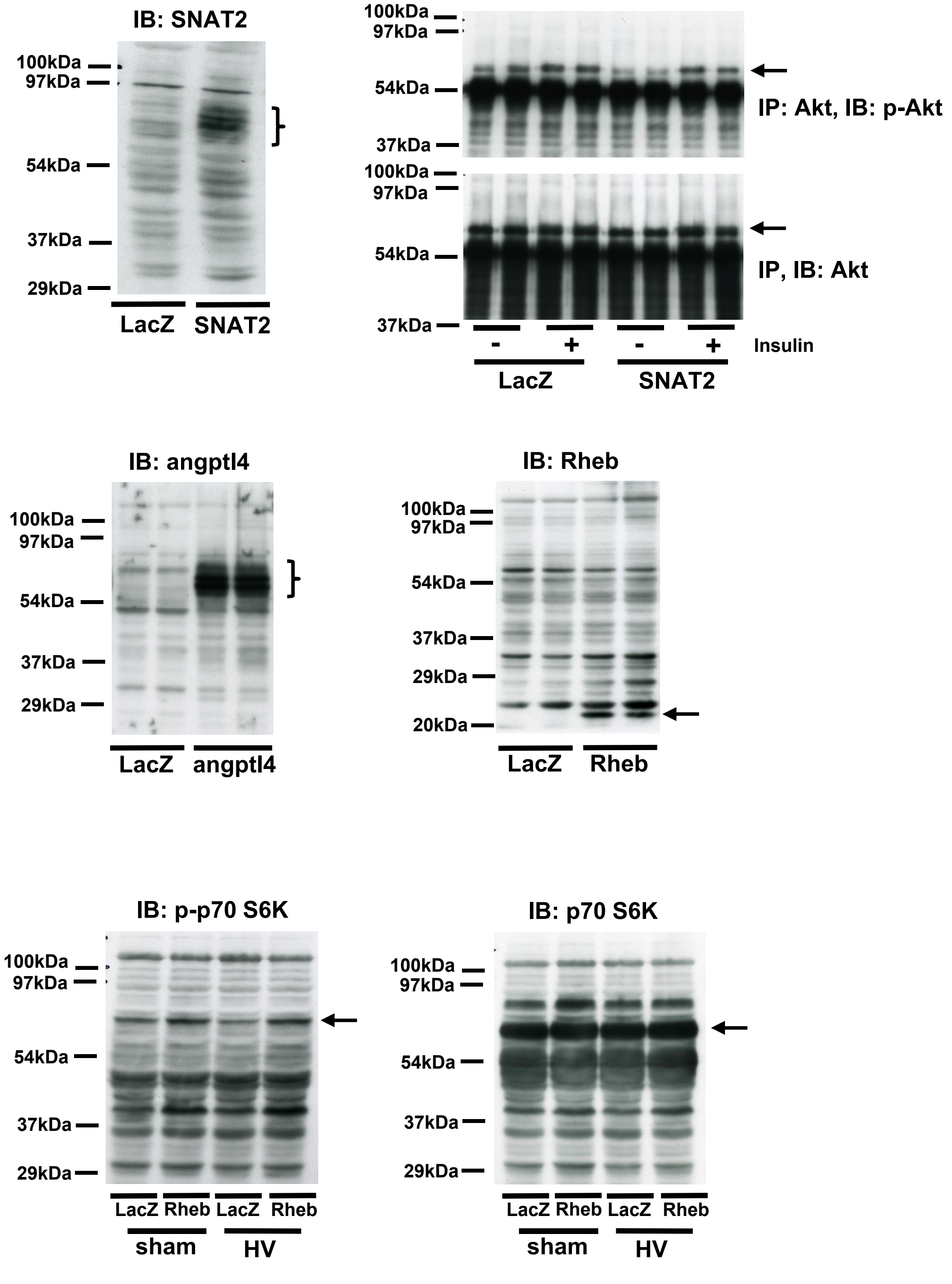

Supplementary Figure 19. Full western blots in Supplementary Figures

amino acids (nmol/mg)	LacZ	SNAT2	*P*
**Taurine**	110.36±7.79	65.92±6.21	< 0.001
**Phosphoethanolamine**	1.40±0.13	2.13±0.24	< 0.001
**Urea**	40.07±7.87	47.28±8.79	N S
**Aspartic acid**	2.40±0.50	5.76±1.87	< 0.005
**Threonine**	1.62±0.25	2.98±0.55	< 0.001
**Serine**	1.47±0.22	2.09±0.18	< 0.005
**Asparagine**	N D	N D	
**Glutamic acid**	14.35±4.42	29.99±6.58	< 0.005
**Glutamine**	17.60±1.57	37.37±2.07	< 0.001
**Proline**	N D	5.09±2.20	
**Glycine**	10.50±1.51	10.06±1.12	N S
**Alanine**	21.42±3.62	38.26±9.92	< 0.01
**Citruline**	N D	N D	
**Valine**	2.63±0.24	15.27±2.09	< 0.001
**Methionine**	N D	1.51±0.48	
**Isoleucine**	1.19±0.17	2.20±0.38	< 0.001
**Leucine**	2.07±0.28	6.16±1.37	< 0.001
**Tyrosine**	0.90±0.05	1.93±0.42	< 0.001
**Phenylalanine**	N D	N D	
**Histidine**	3.37±0.51	5.38±0.74	< 0.001
**Ornitine**	N D	3.85±0.63	
**Lysine**	2.17±0.44	2.69±0.55	N S
**Arginine**	N D	N D	

Supplementary Table 1. Hepatic SNAT2 expression changes hepatic AA concentrations

SNAT2 or LacZ adenovirus was administered to standard high fat diet-fed C57BL/6 mice. Hepatic AA concentrations were measured for 5 days after adenovirus administration (*n*=5). Data are presented as means±SD. *P* value by the unpaired *t* test.

gene name	forward	reverse
**28SrRNA**	5’ -AAGTCCTTCTGACTGAGGCC- 3’	5’ -ATTCCCAAGCAACCCGACTC- 3’
**b-actin**	5’ -TTGTAACCAACTGGGACGATATGG- 3’	5’ -GATCTTGATCTTCATGGTGCTAGG- 3’
**SNAT2**	5’ -ACATAAGGCGTATGGTCTG- 3’	5’ -TACCACAACCCATTTGTATC- 3’
**LPL**	5’ -CCCAGCTCTATCTTGTTAGT- 3’	5’ -TGGGTCACATGATGGAGT- 3’
**HL**	5’ -TGGCCTCATTCTAAAGACC- 3’	5’ -CTCTTTCGATTCAGTCAGTC- 3’
**Rheb**	5’ -GCTTTGGCAGAATCTTGGAA- 3’	5’ -ATCACCGAGCACGAAGACTT- 3’
**UCP1**	5’ -TACCAAGCTGTGCGATGT- 3’	5’ -AAGCCCAATGATGTTCAGT- 3’
**PGC1a**	5’ -ATACCGCAAAGAGCACGAGAAG- 3’	5’ -CTCAAGAGCAGCGAAAGCGTCACAG- 3’
**SREBP1c**	5’-CATGGATTGCACATTTGAAG-3’	5’-CCTGTGTCCCCTGTCTCA-3’
**MTP**	5’ -CAGTTCTCACAGTACCCGTTC- 3’	5’ -CGTTGCACATCTCAGAGTTC- 3’
**LDLR**	5’ -AGGGAATGAGGAGCAGCC- 3’	5’ -GTTCTTCAGCCGCCAGTT- 3’
**PPARa**	5’-GGATGTCACACAATGCAATTCGC-3’	5’-TCACAGAACGGCTTCCTCAGGT-3’
**apoC-2**	5’-GGGCTCCCTCTTAAGTTACTG-3’	5’-ATGCCTGCGTAAGTGCTC-3’
**apoC-3**	5’-CAGCCCCGGACGCTCCTCAC- 3’	5’ -CGACTCAATAGCTGGAGTTG -3’
**GPIHBP1**	5’-TGGAAGCAGGGACAGAGC AC-3’	5’-TGTGGGAGACGAGCGTGATG-3’
**angptl-3**	5’-GGAGGCTCGATGGAGAAT-3’	5’-GCTTGCTGTCTTTCCAGTC-3’
**angptl-4**	5’-AGTCCTGGGAAGCCTACAAG-3’	5’-GGATGGGAAATTGGAGCA-3’
**angptl-8**	5’-CCTCAATGGCGTGTACAG-3’	5’-AGGTGTAAAGCGTCCTCTTC-3’

Supplementary Table 2. Primers used in quantitative RT-PCR
